# Isolation, Identification, and Antibacterial Mechanisms of *Bacillus amyloliquefaciens* QSB-6 and Its Effect on Plant Roots

**DOI:** 10.3389/fmicb.2021.746799

**Published:** 2021-09-16

**Authors:** Yanan Duan, Ran Chen, Rong Zhang, Weitao Jiang, Xuesen Chen, Chengmiao Yin, Zhiquan Mao

**Affiliations:** National Key Laboratory of Crop Biology, College of Horticulture Science and Engineering, Shandong Agricultural University, Shandong, China

**Keywords:** apple replant disease, *Fusarium* spp., *Bacillus amyloliquefaciens*, metabolites, root, identification

## Abstract

Apple replant disease (ARD) is a common problem in major apple planting areas, and biological factors play a leading role in its etiology. Here, we isolated the bacterial strain QSB-6 from the rhizosphere soil of healthy apple trees in a replanted orchard using the serial dilution method. Strain QSB-6 was provisionally identified as *Bacillus amyloliquefaciens* based on its morphology, physiological and biochemical characteristics, carbon source utilization, and chemical sensitivity. Maximum likelihood analysis based on four gene sequences [16S ribosomal RNA gene (16S rDNA), DNA gyrase subunit A (gyrA), DNA gyrase subunit B (gyrB), and RNA polymerase subunit B (rpoB)] from QSB-6 and other strains indicated that it had 100% homology with *B. amyloliquefaciens*, thereby confirming its identification. Flat standoff tests showed that strain QSB-6 had a strong inhibitory effect on *Fusarium proliferatum*, *Fusarium solani*, *Fusarium verticillioides*, *Fusarium oxysporum*, *Alternaria alternata*, *Aspergillus flavus*, *Phoma* sp., *Valsa mali*, *Rhizoctonia solani*, *Penicillium brasilianum*, and *Albifimbria verrucaria*, and it had broad-spectrum antibacterial characteristics. Extracellular metabolites from strain QSB-6 showed a strong inhibitory effect on *Fusarium* hyphal growth and spore germination, causing irregular swelling, atrophy, rupture, and cytoplasmic leakage of fungal hyphae. Analysis of its metabolites showed that 1,2-benzenedicarboxylic acid and benzeneacetic acid, 3- hydroxy-, methyl ester had good inhibitory effects on *Fusarium*, and increased the length of primary roots and the number of lateral roots of *Arabidopsis thaliana* plantlet. Pot experiments demonstrated that a QSB-6 bacterial fertilizer treatment (T2) significantly improved the growth of *Malus hupehensis* Rehd. seedlings. It increased root length, surface area, tips, and forks, respiration rate, protective enzyme activities, and the number of soil bacteria while reducing the number of soil fungi. Fermentation broth from strain QSB-6 effectively prevented root damage from *Fusarium*. terminal restriction fragment length polymorphism (T-RFLP) and quantitative PCR (qPCR) assays showed that the T2 treatment significantly reduced the abundance of *Fusarium* in the soil and altered the soil fungal community structure. In summary, *B. amyloliquefaciens* QSB-6 has a good inhibitory effect on *Fusarium* in the soil and can significantly promote plant root growth. It has great potential as a biological control agent against ARD.

## Introduction

As a widespread agricultural problem, apple replant disease (ARD) is a serious threat to the major fruit-growing regions of the world (Jaffee et al., 1982; Winkelmann et al., 2018). In Washington state, ARD typically results in a $40,000-per-acre reduction in gross returns over a 10-year period (Smith, 1995; Hewavitharana et al., 2019). Chinese apple cultivation area, total output, and export volume rank first in the world, but most orchards are now in an aging period. Due to the limited land resources, it is inevitable to replant the apple trees when the old orchard is renewed, which leads to the occurrence of ARD (Greene, 1915; Willett et al., 1994; Granatstein et al., 2016; Wang et al., 2016). ARD refers to the common management condition in which poor growth of fruit trees occurs after replanting on a site that has previously supported the same or a closely related species; this phenomenon has been broadly termed “replant disease” or “replant disorder” (Mazzola, 1998; Mazzola and Manici, 2012; Nicola et al., 2018). Specific ARD symptoms in apple trees include weak growth, reduced plant height, impaired root system activity, and poor fruit quality (Tewoldemedhin et al., 2011a, b; Weiß et al., 2017; Nicola et al., 2018; Grunewaldt-Stöcker et al., 2019). It is therefore important to explore new measures for the control of ARD.

Apple replant disease has been attributed to a variety of biotic and abiotic causal factors, but current studies suggest that biotic factors such as fungi (*Rhizoctonia*, *Fusarium*, and *Cylindrocarpon*), Oomycetes (*Pythium*, *Humicola*, and *Phytophthora*), and nematodes (*Pratylenchus*) play a leading role in disease development (Utkhede et al., 1992; Van Schoor et al., 2009; Tewoldemedhin et al., 2011a, b; Kelderer et al., 2012; Tilston et al., 2018, 2020; Yim et al., 2020). This has been widely demonstrated in other studies *via* soil pasteurization and the application of biocides (Weller et al., 2002; Garbeva et al., 2004). The pathogens in replanted orchard soils from different regions vary in population characteristics. Previous studies have suggested that *Fusarium* is one of the main pathogens that cause ARD in China (Yin et al., 2017; Wang G. S. et al., 2018; Sheng et al., 2020). Van Schoor et al. (2009) found that *Fusarium*, *Cylindrosporium*, and *Pythium* are the main harmful fungi that contribute to the occurrence of ARD in South Africa. Kelderer et al. (2012) found that *F. solani* and *F. oxysporum* are the main pathogenic fungi associated with the occurrence of ARD in Italy. Currently, the major control strategy for ARD is the use of soil fumigants such as methyl bromide, metam sodium, and chloropicrin (Utkhede and Smith, 2000; Zasada et al., 2010). Although chemical disinfection can effectively control replant diseases, the use of these chemical disinfectants has a number of disadvantages, including difficulty in application, high cost, and potential impacts on the environment and human health (Brown et al., 2000; Leroux, 2003; Chen et al., 2014). Biological control is considered to be an alternative and more sustainable strategy for plant disease control because of its lower cost and environmental friendliness (Podile and Prakash, 1996; Hoitink and Boehm, 1999; Zalila-Kolsi et al., 2016; Chowdhury et al., 2020). Therefore, the development of biological agents is critical for the biological control of ARD.

*Bacillus* is a new class of biological control agent (Nagórska et al., 2007; Fan et al., 2017b) whose metabolism can produce a variety of antibacterial substances (fengycin, surfactin, etc.), enzymes (amylase, protease, etc.), and nutritional factors, thereby effectively inhibiting the reproduction of pathogens and promoting plant growth (Stein, 2005; Cazorla et al., 2007; Frikha-Gargouri et al., 2017; Zhang C. et al., 2018). *Bacillus* also produces resting spores during its growth and development. These have the advantage of resistance to high temperatures, acids, salt, drugs, and radiation. When the spores are exposed to favorable conditions they resume growth as vegetative cells (Hallmann et al., 1999; Piggot and Hilbert, 2004; McKenney et al., 2013; Zhang X. et al., 2014). *Bacillus* has a high reproductive rate, can easily be mass produced and processed, is convenient to transport, and has a high survival rate during storage (Collins and Jacobsen, 2003). Fan et al. (2017a) isolated *B. subtilis* 9407 from healthy apples in an infested orchard; it can produce antifungal compounds such as fengycin and can effectively prevent apple ring rot disease. Mutaz Al-Ajlani and Hasnain (2010) determined that 54 of 118 *Bacillus* strains isolated from soil samples had antagonistic activities toward at least two strains from a panel of pathogenic and non-pathogenic microorganisms. Zhang M. et al. (2016) isolated *Bacillus amyloliquefaciens* IBFCBF-1 that can effectively control Phytophthora blight and promote the growth of pepper. Singh et al. (2008) found that *B. subtilis* BN1 isolated from the chir pine (*Pinus roxburghii*) rhizosphere exhibited strong antagonistic activity toward *F. oxysporum* and *R. solani*. Cavaglieri et al. (2005) found that *B. subtilis* CE1 reduced *F. verticillioides* colonization of the rhizoplane and endorhizosphere at all inoculum levels found with maize roots. Therefore, the use of *Bacillus* is a potential sustainable alternative for the biological control of ARD.

In this study, we isolated bacteria with biological control effects from healthy apple trees in a replanted orchard in which apple trees showed ARD symptoms. We identified the bacterial isolates using biochemical and molecular approaches. We then tested their ability to produce metabolites with antagonistic activities toward fungal strains and characterized their antifungal compounds by column chromatography and GC–MS. Finally, we evaluated the ability of a bacterial fertilizer to control ARD by measuring plant and root growth and characterizing the soil microbial community, with the aim of providing a new method for ARD control.

## Materials and Methods

### Sample Collection

In May 2016, samples were collected from four orchards that were replanted 4–5 years previously at a pre-existing apple orchards (more than 25 years old). Apple trees with ARD symptoms (twig growth retardation or death) were common in the sampled orchards. Six healthy apple trees were randomly selected from each orchard, and roots, stems, leaves, fruits, and rhizosphere soil were collected from the trees using a sterile spatula for a total of 120 samples. The samples were packed in clean, dry, sterile polyethylene bags, which were placed on dry ice in the field and taken to the National Key Laboratory of Crop Biology as soon as possible. The geographic coordinates of the sampled sites are presented in Supplementary Table 1. The sampled orchards primarily used *Malus* × *robusta* (CarriŠre) Rehder as the rootstock, and *Malus pumila* Mill was the main cultivated scion variety.

### Isolation of Biocontrol Bacteria

Bacteria were isolated by the serial dilution method (Filippi et al., 2011), with some modifications. The soil was filtered through a sieve (diameter 3–4 mm) to remove detritus. Five grams of rhizosphere soil were placed in 45 mL of sterilized water and shaken at 180 rpm for 30 min, then allowed to stand for 5 min to make a stock suspension of soil bacteria. The suspension was diluted to 10^–4^, and a 100-μL aliquot of dilution was plated on Luria–Bertani (LB) agar (10 g tryptone, 5 g yeast extract, 10 g NaCl, 15 g agar, pH 7.0) overnight at 37°C. Each treatment was repeated three times. Distinct single colonies were picked and subcultured to purity on LB agar medium. Each purified strain was inoculated into liquid LB medium with constant shaking at 180 rpm for 12 h at 30°C and stored at −80°C in 15% glycerol-containing liquid LB medium (Frikha-Gargouri et al., 2017). Roots, stems, leaves, and fruits were washed with tap water to remove impurities. After natural air drying and rinsing with distilled water three times, the tissues were surface-sterilized in 75% ethanol (v/v) for 30 s, submerged in 1% NaOCl (w/v) for 10 s, and rinsed three times in sterile distilled water (Dongzhen et al., 2020). One hundred microliters of sterile water from the last rinse were spread on LB medium and cultivated for 24 h to verify that the surface disinfection of the plants was complete. The surface-disinfected plants were cut into small pieces with a sterile scalpel, placed in a sterile mortar, and ground in an appropriate amount of sterile PBS buffer. The grinding solution was diluted to 10^–4^, and the bacteria were isolated as described above for the rhizosphere soil.

### Antagonistic Screening and Broad-Spectrum Verification

#### First Screening

The tested pathogens were all maintained in our laboratory (National Key Laboratory of Crop Biology). The pathogens were grown on potato dextrose agar (PDA) for 7 days at 28°C prior to use. A dual-culture test was conducted to examine whether the isolated strains could antagonize the growth of plant fungal pathogens according to the methods described by Yu et al. (2011), with some modifications. A mycelial disk, Φ1.0 cm in diameter, of a pure culture of each fungal pathogen was placed in the center of a PDA plate, and then the isolated strains was inoculated in four symmetrical spots around the mycelium disk. Each treatment was repeated three times. The plates were incubated for 7–10 days at 28°C. The plates were scanned once a day to monitor the formation of an inhibition zone and the growth of fungal pathogens. The width of the inhibition zones were measured and then averaged. The strain showing the strongest antifungal effect was selected for further studies.

#### Screening

Selected bacterial strains were streaked onto LB agar plates, and single colonies were inoculated into LB broth (100 mL in a 250-mL Erlenmeyer flask) and cultivated with constant shaking at 150 rpm for 48 h at 28°C. Each bacterial culture was centrifuged at 10,000 rpm for 20 min to pellet bacterial cells, then the cells were suspended in PBS buffer, and the cell density was adjusted to the required bacterial density [1 × 10^8^ colony forming units (CFU/ml)] (Chen et al., 2014). Freshly prepared bacterial suspension was used for each experiment. Antifungal activity against pathogens was evaluated using the dual culture assay in PDA medium, Fresh mycelial plugs (10 mm diameter) were cut from the margins of each fungal pathogen colony and were transferred to the center of PDA plate. Four pieces of sterile round filter paper containing 2 μL bacterial cell suspension were placed 2.5 cm from the plugs, and sterilized distilled water (SDW) was used as a control according to the methods described by Erdogan and Benlioglu (2010), with some modifications. After culturing *Penicillium brasilianum* and *Albifimbria verrucaria* on the PDA plate for 7 days, the PDA plate was rinsed with sterile distilled water. The density of the conidial suspension was measured using a hemocytometer and the inoculated spore culture was diluted using sterile distilled water to obtain a final concentration of 1 × 10^6^ spores/mL. Add the conidia suspension to the PDA medium, mix and invert the plate, and then perform the antibacterial test. Plates were incubated at 28°C until control plates covered the entire surface. The percentage inhibition was calculated using the formula. I = (C − T)/(C − D) × 100. Where I:% inhibition, C: the colony diameter of the control (mm), T: colony diameter of the treatment (mm), and D: plug diameter (mm). Three replicates of each treatment were performed, and the assays were repeated three times (Yu et al., 2011; Li et al., 2020).

### Identification of Biocontrol Bacteria

#### Morphological Observation

Isolates were cultured on LB agar at 37°C for 24 h, and their morphological traits were observed. Gram’s staining and endospore staining by Schaeffer–Fulton method was performed as described by Benson (2002) and Bartholomew and Mittwer (1950). The shape and size of the bacteria were observed with a Nikon BX-51 fluorescence microscope and an SU-8010 scanning electron microscope.

#### Physiological and Biochemical Characterization

Physiological and biochemical characteristics were assessed according to the methods described in *Bergey’s Manual of Systematic Bacteriology* (2nd edition) and the Common Bacterial Identification Manual (Garrity, 2007; Vos et al., 2011).

#### Characterization With the Biolog GEN III MicroStation System

A Biolog GEN III microplate system (Biolog Inc., Hayward, CA, United States) was used to analyze carbon source utilization and chemical sensitivity of strain QSB-6 as described in Hu et al. (2010).

#### DNA Extraction, PCR Amplification, and Phylogenetic Analysis

Strain QSB-6 was inoculated into liquid LB medium and cultivated at 30°C for 12 h with shaking at 200 rpm/min. Genomic DNA was extracted using the EasyPure Bacteria Genomic DNA Kit (Del Sal et al., 1988). To confirm the species identity of strain QSB-6, we obtained the DNA sequences of the 16S ribosomal RNA gene (16S rDNA), DNA gyrase subunit A (gyrA), DNA gyrase subunit B (gyrB), and RNA polymerase subunit B (rpoB) (Gardes and Bruns, 1993; Yamamoto and Harayama, 1995; Chun and Bae, 2000; Zalila-Kolsi et al., 2016; Somerville et al., 2020). PCR amplification was performed in a final volume of 50 μL that contained 2.0 μL of genomic DNA (20 ng/μL), 1.0 μL of each primer (10 μM), 0.5 μL of PrimeSTAR HS DNA polymerase (2.5 U/μL), 10.0 μL of 5× PrimeSTAR Buffer (Mg^2+^ Plus), 4.0 μL of dNTP Mixture (2.5 mM each), and 31.5 μL of ddH_2_O (Weisburg et al., 1991). PCR amplification was performed in an Applied Biosystems 2720 Thermal Cycler (Applied Biosystems Inc., United States) with an initial denaturation step at 94°C for 5 min, followed by denaturation at 94°C for 40 s, annealing for 40 s, and extension at 72°C for 1 min. Thirty-five cycles of amplification and a final extension at 72°C for 10 min were performed (Zhang Q. et al., 2014). The primers and annealing temperatures are shown in Supplementary Table 2. After the reaction was complete, 3 μL of PCR product were used for 1% agarose gel electrophoresis to confirm the PCR-amplified fragments. Amplified PCR products were purified using a DNA Gel Recovery Kit (AxyPrep, Hangzhou, China) and sequenced on an ABI 3730XL system (Applied Biosystems Inc., United States) at Personalbio Gene Biotech Co., Ltd. (Qingdao, China).

For phylogenetic analysis, 16S rDNA, gyrA, gyrB, and rpoB sequences closely related to our sequences were retrieved from GenBank based on BLAST results from the National Center for Biotechnology Information.^1^ Maximum likelihood (ML) phylogenies were constructed from the sequence datasets using RAxML-HPC2 run on XSEDE (8.2.12) (Stamatakis, 2014) through the CIPRES Science Gateway^2^ to obtain multiple measures of branch support. The parameters Maximum Hours to Run and Number of Patterns were modified according to the dataset, and other parameters were set to default values (Lombard et al., 2019; Medeiros Araújo et al., 2021). The trees were visualized using FigureTree v1.4.3 and Adobe Illustrator CS6.

### The Inhibitory Effect of Extracellular Metabolites on *Fusarium*

#### Preparation of Fermentation Broth

Strain QSB-6 was cultured on LB agar at 37°C for 24 h, and a single colony was inoculated into LB broth (100 mL in a 250-mL Erlenmeyer flask) and grown in a shaker incubator at 180 rpm for 12 h. It was then inoculated into optimized liquid fermentation medium (20 g sucrose, 15 g yeast extract, 1 g MnSO_4_, 2.0 g NaH_2_PO_4_⋅2H_2_O, and 4.0 g Na_2_HPO_4_⋅2H_2_O in 1 L) at a ratio of 5% and grown in a shaker incubator at 200 rpm for 48 h. The cell-free culture filtrate was obtained as described in Azabou et al. (2020) with some modifications. Culture was then centrifuged at 10,000 rpm for 15 min at 4°C. The supernatant was collected and filtrated through 0.22-μm Nylon 66 microporous membrane, and stored at 4°C for later use.

#### Effect of Cell-Free Culture Filtrate on *Fusarium* Hyphae

A thin layer of PDA medium was poured onto a sterile plate, and a sterile glass slide was placed in the center of the plate after the medium had solidified. Then approximately 200 μL of PDA medium was spread onto the glass slide, and a sterile 1-mL pipette tip was used to place a *Fusarium* cake onto the slide. An Oxford cup was placed at each end of the slide; 200 μL of cell-free culture filtrate was added to one cup as the treatment, and sterile distilled water was added to the other cup as the control. After incubating for 3 days, the slide was observed under a Nikon BX-51 fluorescence microscope. The hyphae at the edge of the bacteriostatic area were picked with an inoculation needle and placed in a sterile centrifuge tube that contained 2.5% glutaraldehyde fixative for 24 h. The control area was sampled similarly, and the samples were sent to Keshang Biotech Co., Ltd., for scanning electron microscope observation.

#### Effect of Strain QSB-6 on *Fusarium* Spore Germination

*Fusarium* was cultured on VBC medium (1 g KH_2_PO_4_, 1 g KNO_3_, 0.5 g sucrose, vitamin B1 tablet, vitamin C tablet, and 20 g agar in 1 L) at 28°C for 7 days, rinsed thoroughly with sterile water, and shaken evenly. The density of the conidial suspension was measured using a hemocytometer, and the suspension was diluted with sterile distilled water to a final concentration of 1 × 10^6^ spores/mL (Gabrekiristos et al., 2018). It was mixed with fermentation broth and cell-free culture filtrate on a concave glass slide at a ratio of 1:1 and mixed with sterile water as a control (Rahman et al., 2007). The slide was kept moist and incubated at 28°C for 24 h in order to measure spore germination rate. Spores were scored as germinated if the germ tube length equaled or exceeded half the diameter of the spore. The percentage inhibition was calculated using the formula. I = G/T × 100%. Where I:% spore germination rate, G: the number of germinated spores and T: the total number of spores. The total number of spores under investigation should be greater than 200, three replicates were conducted for each treatment, and the experiment was repeated twice (Li et al., 2021).

#### Effect of Fermentation Broth on *Fusarium* Biomass

As described in Podile and Prakash (1996) and Omar and Abd-Alla (1998), the fermentation broth was mixed with PDB to make a liquid medium with a concentration of 250 μL/mL (50 mL in a 250-mL Erlenmeyer flask). A 1-mL pipette tip was used to obtain a *Fusarium* cake and place it in a triangular flask to serve as the treatment group. The control group was cultured in sterile water and PDB liquid base mix. After culturing on a constant temperature shaker at 28°C and 180 rpm/min for 6, 12, or 24 h, the mycelium was placed on a pre-weighed Whatman No. 1 filter paper to remove the liquid medium from its surface, then dried in a 65°C oven for 24 h and weighed with an electronic balance. These steps were repeated using mixtures of fermentation broth and PDB with concentrations of 20, 100, and 200 μL/mL, and weights were obtained after 24 h of incubation.

#### Stability of Cell-Free Culture Filtrate

Five milliliters of cell-free culture filtrate in a 10-mL centrifuge tube were placed in a constant temperature water bath at 50, 60, 70, 80, 90, or 100°C for 0.5 h to test the thermal stability of the cell-free culture filtrate. The pH of cell-free culture filtrate was adjusted to 1.0, 3.0, 5.0, 7.0, 9.0, 11.0, or 13.0 with 3 M NaOH or 1 M HCl, and the broth was allowed to stand for 2 h to test its acid-base stability. Broth samples were placed under a 20 W UV lamp and irradiated for 0.5, 1.0, 1.5, 2.0, 2.5, or 3.0 h to assess their UV sensitivity. Samples were also placed in a light incubator (4500 ± 500 lx) for 1, 2, 4, 8, 12, 16, or 32 h to test their light stability. Untreated cell-free culture filtrate samples were used as the control group, and each treatment was replicated three times. Antibacterial activity was measured by the filter paper method, and the antibacterial rate was calculated (Hu et al., 2010).

#### The Protective Effect of Strain QSB-6 on Plant Roots

*Malus hupehensis* Rehd. seedlings were selected as the test material. For treatment 1, the roots were soaked in sterile distilled water for 24 h. For treatment 2, the roots were first soaked for 12 h in fermentation broth from strain QSB-6, then treated with a suspension of *Fusarium* spores for 12 h. For treatment 3, the root system was first soaked in sterile distilled water for 12 h, then treated with a suspension of *Fusarium* spores for 12 h. Each treatment was replicated six times. The roots were placed in a sterile centrifuge tube that contained 2.5% glutaraldehyde fixative and sent to Keshang Biotech Co., Ltd., for paraffin sectioning and Periodic Acid Schiff (PAS) staining (Shao et al., 2020).

### Separation and Purification of Metabolites

Twenty-four liters of active fermentation broth were obtained from strain QSB-6 as described above. After centrifugation, the supernatant was obtained, and the fermentation products were extracted using equal volumes of *n*-butanol, ethyl acetate, chloroform, and petroleum ether. After the extract phase was dried over anhydrous sodium sulfate, it was concentrated under reduced pressure using a Rotary Evaporator N-1300D-WB (Tokyo, Japan) to obtain a crude extract. The *in vitro* antibacterial activity of the crude extract was tested by the filter paper method (Frikha-Gargouri et al., 2017), and the organic phase after extraction with ethyl acetate was found to have better antibacterial activity (Supplementary Table 3). After filtering the crude ethyl acetate extract to remove impurities that were insoluble in methanol, the next purification work was performed.

#### Thin-Layer Chromatography

An appropriate amount of ethyl acetate extract was dissolved in a small amount of methanol. The crude active fraction was separated on a GF254 silica gel thin layer chromatography plate (Yantai Ocean Chemical Technology Co., Ltd., China) using methanol:dichloromethane 1:1, 2:1, 3:1, 4:1, or 5:1 (v/v) as the solvent system. After separation, the plate was visualized under UV light at 254and 364 nm, and the Rf values were recorded. The solvent ratio that best resolved the active compounds was determined to be methanol:dichloromethane = 5:1, and the Rf value was ∼0.7. This solvent ratio was used to configure the flow ratio for silica gel column chromatography (Chen et al., 2016; Chowdhury et al., 2020).

#### Silica Gel Column Chromatography Separation

The silica gel powder used in this experiment was 200–300 mesh. After extraction, the sample was mixed with an appropriate amount of silica gel powder and use a Rotary Evaporator N-1300D-WB (EYELA Tokyo Rikakikai, Japan) to spin-evaporate to powder, then loaded into the column. The column was first eluted with methanol:dichloromethane 5:1) as the mobile phase, and the same amount of effluent was collected after the mobile phase flow had completed. The effluent was eluted with a mobile phase of methanol:dichloromethane 1:1) to flush out active components that remained in the silica gel column, and finally the column was cleaned with pure methanol. The collected effluent was concentrated and analyzed by thin-layer chromatography (TLC). After comparing the bands, the effluent could be divided into six components (Supplementary Table 4). *In vitro* antibacterial activity was measured by the filter paper method, and the antibacterial activity of components IV and V was strongest. The activity of components II and VI was relatively weak, and components I and III showed no antibacterial activity. A spore germination assay showed that components IV and V had the best inhibitory effects. A small amount of methanol was added to dissolve component IV and V, the dissolved extracts were passed through a Nylon 66 0.22-μm filter membrane, and the filtered extracts were stored in a refrigerator at 4°C for later use.

#### Gas Chromatography–Mass Spectrometry

The compounds in the active extract were identified by gas chromatography–mass spectrometry (GC–MS) followed by an NIST17 database search. The GC–MS analysis was performed on a GC–MS-QP2010 Plus instrument (Shimadzu, Japan). The peak area normalization method was used to calculate the relative content of each component. The chromatographic conditions were: capillary column HP-5 (60 m length, 0.25 mm ID, 0.25 μm film, 325°C maximum temperature), injection volume 1 μL, carrier gas He (99.999%), column flow rate 1.0 mL/min, splitless injection, program temperature rise, injection port temperature 280°C, column starting temperature 50°C for 2 min, temperature increase to 180°C at 10°C/min, 1 min at 180°C, temperature increase to 270°C at 6°C/min, and 15 min at 270°C. The mass spectrometry conditions were full scan acquisition mode, relative value EMV mode, full scan acquisition mass range 50–550 amu, ion source EI 70 eV, interface temperature 230°C, and ion source temperature 200°C.

#### Analysis of Chemical Composition of Metabolites

Metabolites were analyzed using the test-tube detection method and the TLC chromogenic method described in the experimental methods for quality and chemical composition of traditional Chinese medicines (Zhong et al., 2012).

#### Antibacterial Effect of Pure Product

A conidial suspension of *Fusarium* was obtained as described above and mixed with PDA medium. The plate was inverted, and two Oxford cups were placed on the left and right, one containing 200 μL of sterile distilled water and the other containing 200 μL of pure product (10 μg/L). The plate was incubated at 28°C for 5 days, and the zone of inhibition was measured as described in Zhang et al. (2013), with some modifications. Effect of 1,2-benzenedicarboxylic acid on mycelial growth of plant fungal pathogens was evaluated as the description of Li et al. (2021) with some modifications. Briefly, 1,2-benzenedicarboxylic acid with methanol were diluted to different concentrations (10, 50, 100, and 1000 μg/L) and added to the sterilized PDA medium, respectively. Equal amount of methanol was used as a control. A 5 mm-diameter fungal disc was placed on the plate center. Plates were incubated at 28°C until control plates covered the entire surface. The percentage inhibition was calculated using the above formula. Basic information on the pure product is provided in Supplementary Table 5.

#### Plant Growth Promotion Activities of Pure Product

The plant growth promotion activities of the two compounds were measured by the modified method described by Wu et al. (2019). Briefly, three 2−day−old germinated *Arabidopsis thaliana* Col−0 seedlings were transferred to the one side of the I−plate containing 0.5 × MS, 0.8% sucrose, and 1% Bacto agar. Then, the two synthetic compounds were diluted separately in ethanol, and 20 μL of the resulting suspension was applied to a sterile filter paper disk on the other side of the I−plate. A total of 10, 100, 500, and 1000 μg doses of each pure product were tested. Each treatment was repeated for three times. The fresh weight of the *A. thaliana* Col−0 seedlings was measured after 10 days.

### Field Experiments

#### Test Material

Soil from a 31-year-old apple orchard was collected in Xiaowangzhuang Village, Manzhuang Town, Taian, China (Lon: 117.081039, Lat: 36.06682). Soil samples were collected 80 cm away from the trunk and 20–40 cm from the soil surface. Samples were collected randomly at multiple points and mixed. The basic soil conditions are shown in Supplementary Table 6. Microbial fertilizer was produced by Chuangdi Microbial Resources Co., Ltd., Dezhou, China. The bacterial manure carrier was cow manure: straw (3:1), and the bacterial density was 2.1 × 10^9^ CFU per gram. The available nitrogen content was 0.36 mg⋅g^–1^, available phosphorus was 1.49 mg⋅kg^–1^, and available potassium was 1.03 mg⋅kg^–1^.

#### Pot Experiment

The pot experiment was performed at the National Apple Engineering Experiment Center of the Horticultural Science and Engineering College of Shandong Agricultural University and the State Key Laboratory of Crop Biology (Lon: 117.156540, Lat: 36.164443). In March 2017, *Malus hupehensis* Rehd. seedlings were transplanted to the trays. When the seedlings had grown a third true leaf, seedlings of similar growth potential were transplanted into clay pots (38 cm × 28 cm × 26 cm). Each pot contained 75.43 kg of soil, and there were two seedlings per pot and 20 pots in each treatment group. The pots were divided into the following treatments: untreated soil from a 31-year-old orchard (CK1), the same soil fumigated with methyl bromide (CK2), the same soil treated with the manure carrier only (T1), and the same soil treated with the *B. amyloliquefaciens* strain QSB-6 manure treatment (T2). The application amount of bacterial fertilizer and manure carrier accounted for about 1% of the soil weight (Ma et al., 2018). All experiments were performed with normal water and manure management. Soil samples were collected from three randomly selected pots per treatment on July 15, August 15, and September 15, 2017. Soil was removed around the surface layer and basin, and three basins for each treatment were randomly selected as three replicates. Impurities were removed using a 2-mm sieve, and the soil samples were divided and stored in two sealed pockets. One sealed pocket was stored in a refrigerator at 4°C for measurement of soil microorganisms; the other was stored at −80°C for extraction of soil DNA, quantitative PCR (qPCR), and terminal restriction fragment length polymorphism (T-RFLP) analysis. Three seedlings per treatment were harvested and washed for biomass measurement in the laboratory.

#### Measurement Indices

##### Soil physical and chemical properties

Organic matter, nitrogen, phosphorus, and potassium contents were measured as described in the third edition of *Agrochemical Analysis of Soils* by Bao Shidan (Bao, 2000). Soil pH was measured using a soil to water ratio of 1:2.5 (w/v) with a PHS-3E digital pH meter (LEICI, Shanghai). Soil particle size distribution (the percentage of clay, silt, and sand) was determined by the hydrometer method (Avery, 1973).

##### Microbiological culture

Referring to the method of Zhang Q. et al. (2014), the populations of soil microbes (bacteria, fungi, and actinomycetes) were assessed using the dilution method of plate counting. The bacteria, fungi, and actinomycetes were incubated with beef broth peptone substrate, PDA (Difco), and Gause No. 1 substrate, respectively. Five plates per dilution were measured for each parameter for each soil sample (Guo et al., 2015; Wang et al., 2015). The CFUs per gram of dry soil was used as the unit of the populations of bacteria, fungi, and actinomycetes.

##### Biomass and related parameters

The height and ground diameter of *M. hupehensis* Rehd. seedlings were measured with a rice meter and a vernier caliper (Sangon Biotech, Shanghai); fresh and dry weights were measured with an electronic balance (OLABO, China).

##### Root-related indices

A Microtek ScanMaker i800 Plus scanner (Shanghai Zhongjing Technology Co., Ltd., China) was used to scan the root system. A Wanshen LA-S series plant root analysis system was used to process the sample images; total root length, surface area, forks, and tips were measured. Respiratory rate was measured by the methods of Gao et al. (2010). Roots of uniform diameter were selected, cut into 2-mm lengths (∼0.1 g), and placed in buffer in the measurement cuvette of the OXY-LAB oxygen electrode system (Hansatech, United Kingdom) at 25°C for respiration measurement.

##### Root protective enzyme activity

Superoxide dismutase (SOD) activity was measured as described in Zhang et al. (2009), peroxidase (POD) activity as described in Omran (1980), catalase (CAT) activity as described in Singh et al. (2010), and malondialdehyde (MDA) content as described in Hu et al. (2012).

##### DNA extraction and quantitative PCR of *Fusarium*

For each replicate pot described above, a 5 g soil sample was obtained and DNA was extracted using the PowerMax soil DNA isolation kit (MO BIO Laboratories Inc., Carlsbad, CA, United States). The quality and quantity of DNA was determined using an EPPENDORF BioPhotometer nuclei acid and protein analyzer (Eppendorf, Germany). The abundance of *Fusarium* was quantified by qPCR following a modified method (Zhao et al., 2014; Huang et al., 2015; Shen et al., 2018). The primers and annealing temperatures are presented in Supplementary Table 2. Quantitative PCR amplifications for standard and environmental DNA samples were performed with a total volume of 20 μL in each reaction using the SYBR^®^ Premix Ex Taq^TM^ (TaKaRa, Japan) and a CFX96 Touch^TM^ Real-Time PCR Detection System (Bio-Rad, United States). Each PCR reaction contained 2 μL of the target DNA, 10 μL of SYBR Green premix Ex Taq, 0.4 μL of each primer, and 7.2 μL sterile distilled water. Thermal cycling conditions consisted of 30 s at 95°C followed by 40 amplification cycles of 5 s at 94°C, annealing for 30 s, and extension at 72°C for 1 min. Each sample was performed with three parallels, and the results were expressed as log copy numbers per gram of dry soil.

##### Terminal-restriction fragment length polymorphism analysis

Terminal restriction fragment length polymorphism analysis was used as a tool to rapidly and qualitatively compare fungal community structure across the different treatments. DNA was amplified using the universal primers ITS1F-FAM/ITS4R (Supplementary Table 2) that target the fungal ITS region between the 18S and 28S rRNA regions, respectively. The forward primers were labeled at the 5’end with 6-carboxyfluorescein (FAM) which was synthesized by Sangon Biotech (Shanghai, China). The specific steps refer to the method of Quéric and Soltwedel (2012) and Xu et al. (2019). The 50 μL PCR mixture contained 0.6 μL of 5 U/uL of Ex Taq (TaKaRa, Japan), 5 μL of 10× Ex Taq Buffer, 1 μL of 2.5 mM dNTP mixture, 2 μL of 0.5 mM of forward and reverse primer, 12.6 μL of ddH_2_O and 2.0 μL of 100 ng of the extracted DNA as template. All PCR amplification was carried on an Applied Biosystems 2720 Thermal Cycler (Applied Biosystems Inc., United States). The PCR conditions consisted of 5 min at 95°C, followed by 30 cycles of denaturing at 94°C for 30 s, annealing at 50°C for 30 s, and extension at 72°C for 1 min, and a final 10 min extension at 72°C. PCR products were cleaned by EZNA PCR purification kit (Omega Bio-Tek, United States), following the manufacturer’s instruction and quantified using a DNA master nuclei acid and protein analyzer (Dynamica, United Kingdom). The enzyme reaction system for enzyme digestion was 17.5 μL of purified PCR product (500 ng), 2 μL of Buffer, and 0.5 μL of *Hin*fI enzymes (TaKaRa, Japan) at 37°C in the dark for 6 h (Yu et al., 2014; Zhang Q. X. et al., 2018), and sequenced by Sangon Biotech Co., Ltd., China.

### Data Analysis

Statistical analyses were performed using IBM SPSS 20.0 (IBM SPSS Statistics, IBM Corporation, United States) *via* a one-way analysis of variance (ANOVA). The figures were plotted with Microsoft Excel 2013 (Microsoft Corporation) and Graphpad prism 7.0 (GraphPad software, Inc., United States). The standard deviation (SD) was illustrated using an error bar. The significant differences among treatment-groups and controls were illustrated using the different letters above the columns at the *p* < 0.05 level *via* the least significant difference (LSD) test.

Terminal restriction fragment length polymorphism profiles were analyzed using the Peak Scanner^TM^ Software v1.0 (Thermo Fisher Scientific, Wilmington, United States) with an exclusion of, T-RFs less than 50 bp in length or contributing less than 0.5% of peak area in each sample for subsequent analyses. The apparent T-RFs sizes in capillary electrophoresis are compared against MiCA database to analysis the phylotype. The R statistical platform (v.4.1.1) was used for principal coordinates (PCoA) and cluster analysis to study the difference of sample community composition. The differences between samples were calculated by Bray–Curtis, and analysis of similarity (ANOSIM) was conducted to identify the significant differences between the fungal communities (Liu et al., 2019; Xu et al., 2020). Richness index (SR) and evenness index (E) were calculated by Bio-dap software were calculated by Bio-dap software (Fierer et al., 2003; Jun and Dongyan, 2004; Shyu et al., 2007; Yuan et al., 2007; Percent et al., 2008).

## Results

### Isolation and Validation of Biocontrol Bacteria

Based on colony morphology, size, color, smoothness, and other properties, 509 bacterial strains of differing morphologies were isolated from healthy apple trees in replanted orchards. Antibacterial testing was performed by the flat stand-off and filter paper methods, and 256 strains of biocontrol bacteria were re-screened (Supplementary Table 1). The strain QSB-6 showed a particularly strong broad-spectrum inhibitory effect, and therefore, this strain was selected for further study.

### Identification of Strain QSB-6 by Morphological Observation

The strain QSB-6 was cultured on LB agar at 37°C for 24 h; the surface of the colony was opaque and yellowish and showed the formation of folds (Supplementary Figure 1A). It can form biofilms when grown in liquid medium. The bacteria exhibited a blue–purple color after Gram staining and were identified as Gram-positive. When observed under a fluorescence microscope (100×/1.30 oil lens), individual bacteria were short and rod-shaped; they formed ovoid spores (0.6–0.8 × 1.0–1.4 μm), mesophytic, or subterminal, and the cyst was not enlarged (Supplementary Figures 1B,C). Rod-shaped cells of approximately 0.7–0.9 × 1.8–3.0 μm were observed under an SU-8010 scanning electron microscope (Supplementary Figures 1D–I).

### Carbon Source Utilization and Chemical Sensitivity Assays

The strain QSB-6 was able to utilize D-fructose, D-cellobiose, sucrose, α-D-glucose, gelatin, L-lactic acid, and citric acid as carbon sources. It was sensitive to pH 5, pH 6, 1% NaCl, 4% NaCl, 8% NaCl, 1% sodium lactate, and sodium butyrate (Supplementary Table 7). Physiological and biochemical tests showed that strain QSB-6 could produce hydrogen peroxide and hydrogen sulfide, hydrolyze starch, and reduce nitrate. Contact enzyme, arginine dihydrolase, methyl red reaction, Voges–Proskauer reaction, and gelatin hydrolysis enzyme tests were positive, whereas indole enzyme and urea enzyme reaction tests were negative. According to *Bergey’s Manual of Systematic Bacteriology* (2nd edition) and the Common Bacterial Identification Manual, the physiological and biochemical properties of strain QSB-6 matched those of *B. amyloliquefaciens* (Supplementary Table 8).

### Phylogenetic Analysis

**FIGURE 1:**
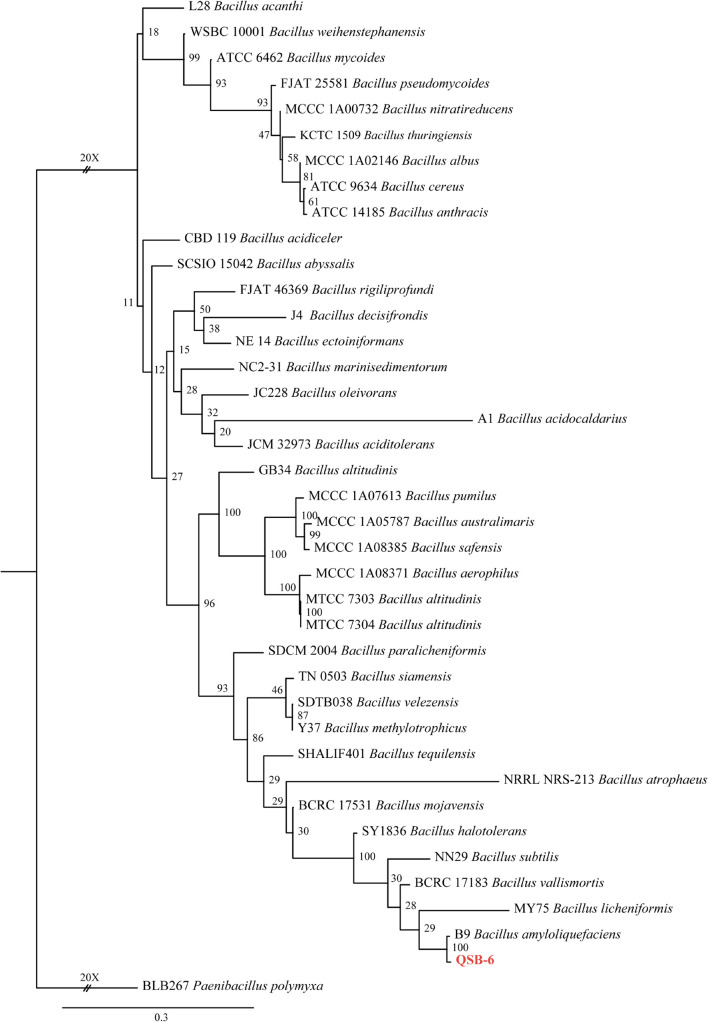
The ML consensus tree inferred from the combined 16S rDNA, gyrA, gyrB, and rpoB sequence alignment. Support for each branch in the inferred tree was evaluated using 1000 bootstrap replications. Support values (ML bootstrap and posterior probability values) are indicated at the branches. The scale bar indicates 0.3 expected changes per site. Clade numbers and Latin name are provided on the right of the tree and these are used for reference in the treatment of the species. The tree is rooted to *Paenibacillus polymyxa* (BLB267). Strains QSB-6 are indicated in bold and red.

Approximately 554–929 bases were sequenced for rpoB and gyrA, 1452 bases for 16S rDNA, and 1066 bases for gyrB. Congruency analysis revealed no conflict between the 16S rDNA, gyrA, gyrB, and rpoB sequences, and the sequences were therefore combined. The combined sequence dataset included 38 ingroup taxa, with *Paenibacillus polymyxa* (BLB267) as the outgroup taxon. ML analysis of identities based on the four gene sequence alignments revealed that strain QSB-6 had the highest homology with *B. amyloliquefaciens* (Figure 1). In summary, strain QSB-6 was identified as *B. amyloliquefaciens*.

### Effects of Strain QSB-6 on the Spore Germination and Hyphae of Fungal Pathogens

**FIGURE 2:**
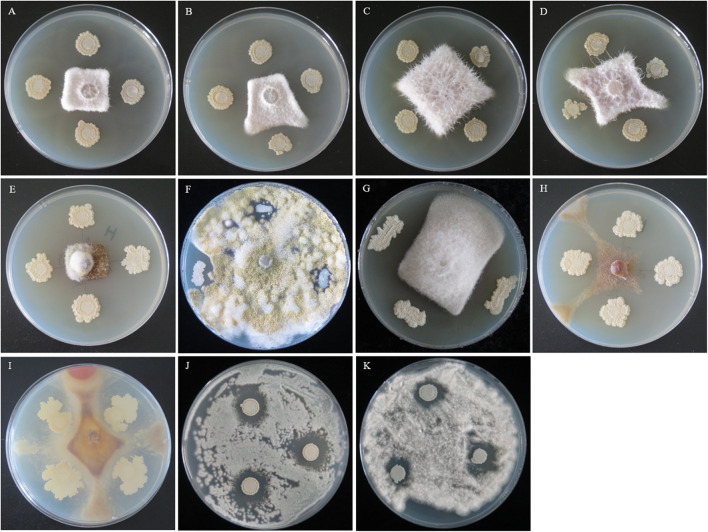
The flat standoff test between strain QSB-6 and pathogenic fungi (PDA medium). **(A)** *Fusarium proliferatum*, **(B)** *Fusarium solani*, **(C)** *Fusarium verticillioides*, **(D)** *Fusarium oxysporum*, **(E)** *Alternaria alternata*, **(F)** *Aspergillus flavus*, **(G)** *Phoma* sp., **(H)** *Valsa mali*, **(I)** *Rhizoctonia solani*, **(J)** *Penicillium brasilianum*, and **(K)** *Albifimbria verrucaria*.

**FIGURE 3:**
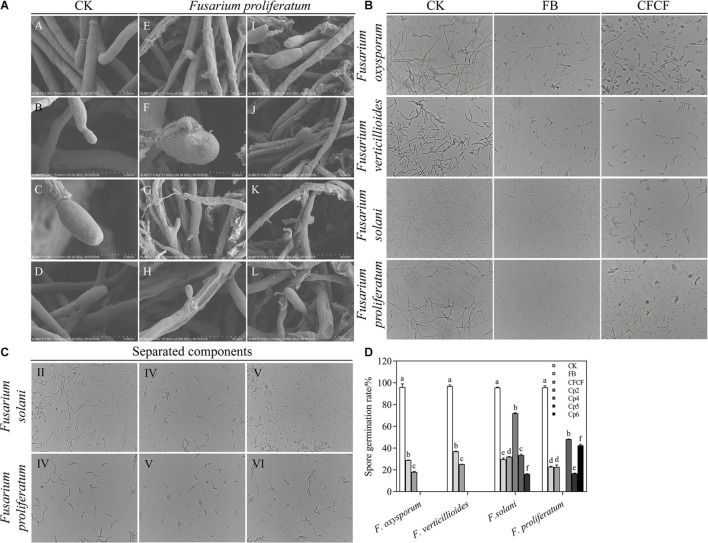
**(A)** The mycelia and spore morphology of *Fusarium proliferatum* under the scanning electron microscope. **(A–D)** Was the normal mycelium and spore, **(E–L)** was the mycelium treated with cell-free culture filtrate. The mycelium showed irregularly reticulated and uneven in thickness **(E,G,J)**, shrinkage **(E–L)**, twisted **(K,L)**, thinned **(E,I–L)**, broken **(E–L)**, the spore cell wall was broken and deformed **(H–L)**, and the mycelium were dissolved, ruptured and overflowed cell contents **(J–L)**. **(B)** The effect of strain QSB-6 on the spore germination of *Fusarium*. CK *Fusarium* spore suspension was mixed with sterile water at 1:1, FB *Fusarium* spore suspension was mixed with fermentation broth at 1:1, CFCF *Fusarium* spore suspension was mixed with cell-free culture filtrate at 1:1. **(C)** The effect of separated components of fermentation broth on spore germination of *Fusarium solani* and *Fusarium proliferatum*. **(D)** Spore germination rate of different treatments, Cp2, Cp4, Cp5, Cp6 represent the separated substances. Lowercase letters above the columns indicate a significant difference at *p* < 0.05. Values are mean ± SD.

Strain QSB-6 had a strong inhibitory effect on the mycelial growth and spore germination of *Fusarium* (Figures 2, 3). The inhibitory rate of strain QSB-6 against *F. proliferatum* was highest, reaching 87% in the PDA plate. The spore germination rate after treatment with fermentation broth and cell-free culture filtrate was 76% lower than that of the control (Table 1 and Figure 3). QSB-6 also had an inhibitory effect on *F. oxysporum*, *F. verticillioides*, and *F. solani*, with inhibition rates of 74, 73, and 84%, and the spore germination rate after treatment dropped by more than 60% compared with the control (Table 1 and Figure 3). The biocontrol bacteria QSB-6 also had an inhibitory effect on 7 common pathogenic fungi, and the inhibitory effect was greater than 48% (Table 1). In particular, the inhibition rate of *A. alternata* reached 87%, followed by the inhibition rates of *V. mali*, *R. solani*, and *Phoma* sp., which also reached greater than 68%. All pathogens exhibited an inhibition zone at the colony junction. These preliminary results showed that strain QSB-6 had a broad-spectrum inhibitory effect on plant pathogens and underscored its potential for development as a broad-spectrum agricultural biocontrol agent.

**TABLE 1:** The inhibitory effect of strain QSB-6 on 11 kinds of plant pathogens.

Treatment	Colony diameter (cm)	Inhibition zone (mm)	Inhibition rate (%)^1^	Inhibition rate (%)^2^
*Fusarium proliferatum*	0.60 ± 0.01f	+++	86.74 ± 0.15a	22.96 ± 0.74abc
*Fusarium verticillioides*	1.22 ± 0.01c	++	72.89 ± 0.13d	19.26 ± 4.12bc
*Fusarium oxysporum*	1.17 ± 0.01c	++	74.00 ± 0.13d	12.59 ± 0.74d
*Fusarium solani*	0.71 ± 0.01e	+++	84.22 ± 0.26b	18.52 ± 2.96c
*Alternaria alternata*	0.60 ± 0.01f	+++	86.74 ± 0.20a	25.19 ± 0.74ab
*Phoma* sp.	1.41 ± 0.01b	+++	68.59 ± 0.27e	27.41 ± 1.48a
*Valsa mali*	0.65 ± 0.02ef	+++	85.48 ± 0.27ab	20.74 ± 1.96bc
*Rhizoctonia solani*	1.02 ± 0.07d	++	77.33 ± 0.51c	11.85 ± 0.74d
*Aspergillus flavus*	−	++	−	7.41 ± 1.96d
*Penicillium brasilianum*	−	++	−	11.11 ± 1.28d
*Albifimbria verrucaria*	−	+	−	7.41 ± 0.74d

*Values are mean ± SD.*

*–, no inhibition zone; +, weak inhibition with inhibition zone <5 mm, growth of the fungus was stopped at the bacterial-streak line; ++, moderate inhibition with inhibition zone 5–10 mm; +++, strong inhibition with inhibition >10 mm. Different letters indicate significantly different at 5% level by Duncan’s new multiple range test. ^1^Inhibition rate = (control colony radius − treatment colony radius)/control colony radius × 100%. ^2^Inhibition rate = Inhibition zone width/control colony radius × 100%.*

### Effect of Strain QSB-6 Cell-Free Culture Filtrate on *Fusarium* Hyphae

The control *Fusarium* mycelium was uniform in thickness and slender with fewer branches; it was full of spores, exhibited a complete structure, and appeared to be in a good growth state (Supplementary Figures 2A,B and Figures 3A, A–D). The mycelium treated with the cell-free culture filtrate was irregularly reticulated, swollen, and uneven in thickness; its hyphae appeared to be curved at the tip, twisted, thinned, broken, shrunken, and shriveled. The mycelia were dissolved and ruptured, and their cell contents had overflowed (Supplementary Figures 2C–H and Figures 3A, E–L). The dry weight of *Fusarium* hyphae decreased significantly in response to treatment with fermentation broth and, was reduced by 55.87, 58.45, and 81.14% after 6, 12, and 24 h of treatment relative to the control group (Supplementary Figure 3). As the concentration of fermentation broth increased, the mycelial dry weight decreased significantly and then stabilized. These results indicated that metabolites produced by QSB-6 during fermentation affected the normal growth of *Fusarium* mycelium.

### Stability of Cell-Free Culture Filtrate

As the temperature of the cell-free culture filtrate rose, its antibacterial rate clearly decreased (Supplementary Figure 4A), perhaps owing to the denaturation and loss of biological activity of antibacterial substances (proteins, enzymes, etc.) in the broth under high temperatures. As pH increased, the antibacterial rate first increased and then decreased; it was close to the antibacterial rate of the control group at pH 7 (Supplementary Figure 4C). The physiological activity of the antibacterial substance(s) in the cell-free culture filtrate may have been inhibited under acidic or alkaline conditions. The antibacterial rate of the cell-free culture filtrate after UV irradiation or exposure to a light intensity of 4500 ± 500 lx did not differ from that of unirradiated broth and always remained above 70% (Supplementary Figures 4B,D). The antagonistic active substance in the cell-free culture filtrate was therefore not sensitive to light.

### Partial Characterization of Antifungal Compounds

**FIGURE 4:**
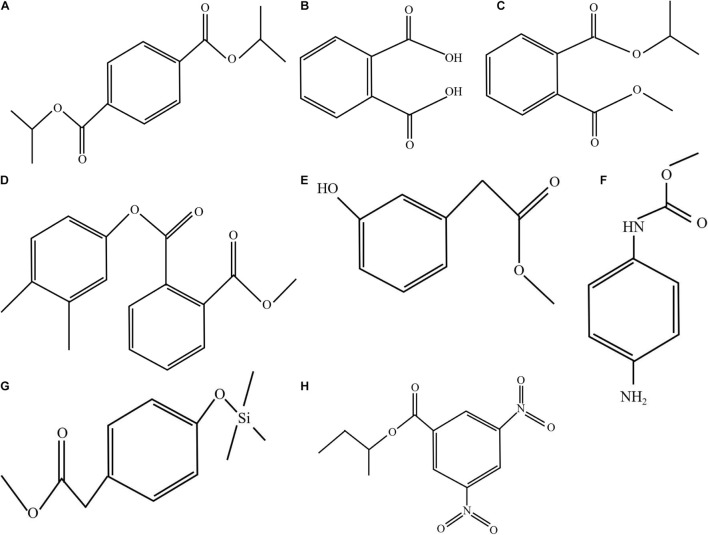
Molecular structure of the representative compounds. Mass spectrum of the compound obtained by GC–MS analysis was compared with the NIST17 spectral database. **(A)** Terephthalic acid, diisopropyl ester (GC RT 28.92, 30.022, 29.634, and 29.835 min); **(B)** 1,2-benzenedicarboxylic acid (RT 22.28 and 23.058 min); **(C)** isopropyl methyl phthalate (RT 26.132, 26.542, and 27.051 min); **(D)** phthalic acid, 3,4-dimethylphenyl methyl ester (RT 27.647 min); **(E)** benzeneacetic acid, 3- hydroxy-, methyl ester (RT 27.122, 26.117, 31.463, and 31.223 min); **(F)** carbamic acid (4-aminophenyl)-, methyl ester (RT 26.698 and 25.615 min); **(G)** methyl 4-hydroxyphenylacetate, TMS derivative (RT 25.895); and **(H)** sec-butyl 3,5-dinitrobenzoate (RT 27.55 min).

**FIGURE 5:**
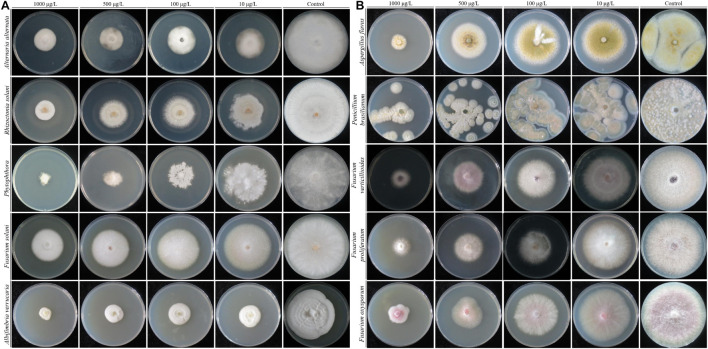
Growth inhibition of plant fungal pathogens on the PDA medium after treated with different gradient 1,2-benzenedicarboxylic acid. **(A)** The inhibitory effect on the growth inhibition of *Alternaria alternata, Rhizoctonia solani, Phytophthora, Fusarium solani* and *Albifimbria verrucaria*. **(B)** The inhibitory effect on the growth inhibition of *Aspergillus flavus*, *Penicillium brasilianum*, *Fusarium verticillioides, Fusarium proliferatum* and *Fusarium oxysporum*.

The results obtained by the test tube method and by thin layer chromatography were essentially the same, confirming the accuracy of the analysis results. Metabolites produced by strain QSB-6 consisted of steroid, lactones, coumarins and their glycosides, flavonoids, cardiac glycosides, amino acids, peptides and proteins, sugars, polysaccharides, phenols, alkaloids, organic acid, and saponin (Supplementary Tables 9, 10). After chromatographic separation, components IV and V were found to have a significant inhibitory effect on the growth of pathogenic fungi and the germination of *Fusarium* spores (Figures 3C,D and Supplementary Figure 5). After GC–MS chromatographic detection and analysis, the main antibacterial substances with Area% >1.0 and retention index RI >1000 was selected from component V (Table 2 and Supplementary Figure 10), and the main antibacterial substances with Area% >0.5 and retention index RI >1000 was selected from component IV (Table 3 and Supplementary Figure 10). Most substances identified from the two components were organic acids and esters. NIST spectral database analysis of the compounds’ mass spectra identified them as terephthalic acid, 1,2-benzenedicarboxylic acid, isopropyl methyl phthalate, phthalic acid, benzeneacetic acid, carbamic acid, methyl 4-hydroxyphenylacetate, sec-butyl 3,5-dinitrobenzoate, 4-(2-methoxyethyl)phenol (Figure 4). The mass spectra of the compounds are shown in Supplementary Figure 6. An *in vitro* antibacterial test demonstrated that terephthalic acid, diisopropyl ester, 1,2-benzenedicarboxylic acid, carbamic acid (4-aminophenyl)-, methyl ester, and benzeneacetic acid, 3- hydroxy-, methyl ester had varying degrees of inhibitory effect on *Fusarium*, among which 1,2-benzenedicarboxylic acid has the best inhibitory effect on *Fusarium*, and as the concentration increases, the inhibitory effect on 11 strains of pathogenic fungi was stronger (Figure 5 and Supplementary Figure 7). At a concentration of 1000 μg/L, the inhibition rates of *F. proliferatum*, *F. verticillioides*, *F. oxysporum*, and *F. solani* were 77.74, 65.52, 68.85, and 47.61%, respectively (Supplementary Table 11). Of the four pure compounds, visual inspection revealed that plant shoot and root growth, when compared with the control treatment (water and ethanol), was only enhanced by 1,2-benzenedicarboxylic acid (100 μg) and benzeneacetic acid, 3- hydroxy-, methyl ester (500 μg). The length of primary root of Arabidopsis seedlings was increased approximately 1.50−fold by the presence of 1,2-benzenedicarboxylic acid and 4.03−fold by benzeneacetic acid, 3- hydroxy-, methyl ester, compared to ethanol treatment, respectively (Supplementary Figure 9). These results indicate that the principle antifungal compounds produced by strain QSB-6 are organic acid esters with benzene ring. It plays important roles in disease control and in growth promoting processes.

**TABLE 2:** Gas chromatography–mass spectrometry identification result of component V.

Number	Retention time (min)	Area%	Ingredient name	Molecular formula	Molecular weight	Retention index	CAS number
1	28.92	23.34	Terephthalic acid, diisopropyl ester	C14H18O4	250	1710	6422-84-0
2	29.45	9.51	Terephthalic acid, isopropyl propyl ester	C14H18O4	250	1774	0-00-0
3	30.022	7.77	Terephthalic acid, diisopropyl ester	C14H18O4	250	1710	6422-84-0
4	22.28	7.28	1,2-Benzenedicarboxylic acid	C8H6O4	166	1620	88-99-3
5	29.634	6.57	Terephthalic acid, diisopropyl ester	C14H18O4	250	1710	6422-84-0
6	29.13	5.88	Terephthalic acid, isopropyl propyl ester	C14H18O4	250	1774	0-00-0
7	29.754	3.43	Terephthalic acid, isopropyl propyl ester	C14H18O4	250	1774	0-00-0
8	29.835	2.93	Terephthalic acid, diisopropyl ester	C14H18O4	250	1710	6422-84-0
9	23.058	2.71	1,2-Benzenedicarboxylic acid	C8H6O4	166	1620	88-99-3
10	26.132	2.06	Isopropyl methyl phthalate	C12H14O4	222	1575	71369-19-2
11	27.647	1.69	Phthalic acid, 3,4-dimethylphenyl methyl ester	C17H16O4	284	2240	0-00-0
12	26.542	1.58	Isopropyl methyl phthalate	C12H14O4	222	1575	71369-19-2
13	27.255	1.4	Benzeneacetic acid, 4- hydroxy-, methyl ester	C9H10O3	166	1380	14199-15-6
14	27.051	1.28	Isopropyl methyl phthalate	C12H14O4	222	1575	71369-19-2
15	22.79	1.24	Phthalamic acid	C8H7NO3	165	1673	88-97-1

**TABLE 3:** Gas chromatography–mass spectrometry identification result of component IV.

Number	Retention time (min)	Area%	Ingredient name	Molecular formula	Molecular weight	Retention index	CAS number
1	26.698	7.78	Carbamic acid (4-aminophenyl)-, methyl ester	C8H10N2O2	166	1571	6465-03-8
2	28.604	7.6	Benzeneacetic acid, 4-(acetyloxy)-, methyl ester	C11H12O4	208	1540	35400-15-8
3	27.122	7.36	Benzeneacetic acid, 3- hydroxy-, methyl ester	C9H10O3	166	1380	42058-59-3
4	25.3	7.14	Benzeneacetic acid, 4- hydroxy-, methyl ester	C9H10O3	166	1380	42058-59-3
5	31.69	5.54	Benzeneacetic acid, 4-hydroxy-	C8H8O3	152	1470	156-38-7
6	25.895	5.46	Methyl 4-hydroxyphenylacetate, TMS derivative	C12H18O3Si	238	1458	27798-62-5
7	25.615	5.05	Carbamic acid (4-aminophenyl)-, methyl ester	C8H10N2O2	166	1571	6465-03-8
8	29.427	4.48	Benzeneacetic acid, 4-hydroxy-	C8H8O3	152	1470	156-38-7
9	26.308	3.59	Phenol, 4-(1-piperidin-1-ylcyclohexylmethyl)-	C18H27NO	273	2360	0-00-0
10	29.078	3.32	Benzeneacetic acid, 4-hydroxy-	C8H8O3	152	1470	156-38-7
11	26.117	3.1	Benzeneacetic acid, 3- hydroxy-, methyl ester	C9H10O3	166	1380	42058-59-3
12	26.825	2.57	Benzeneacetic acid, 3-(acetyloxy)-, methyl ester	C11H12O4	208	1540	35400-14-7
13	27.55	2.19	sec-Butyl 3,5-dinitrobenzoate	C11H12N2O6	268	2085	0-00-0
14	29.356	1.94	4-(2-Methoxyethyl)phenol	C9H12O2	152	1289	56718-71-9
15	27.739	1.88	Benzeneacetic acid, 4-hydroxy-	C8H8O3	152	1470	156-38-7
16	31.463	1.46	Benzeneacetic acid, 3-hydroxy-	C8H8O3	152	1470	621-37-4
17	32.959	1.27	(3S,5R,8aR)-3-butyl-5-methyloctahydroindolizine	C13H25N	185	1454	94535-27-0
18	31.223	1.08	Benzeneacetic acid, 3-hydroxy-	C8H8O3	152	1470	621-37-4
19	31.6	0.92	Benzeneacetic acid, 4-hydroxy-	C8H8O3	152	1470	156-38-7
20	29.871	0.81	Benzeneacetic acid, 3-hydroxy-	C8H8O3	152	1470	621-37-4
21	30.082	0.81	Benzeneacetic acid, 4-hydroxy-	C8H8O3	152	1470	156-38-7
22	30.553	0.81	Methyl mandelate	C9H10O3	166	1322	4358-87-6
23	30.66	0.81	Benzeneacetic acid, 4-hydroxy-	C8H8O3	152	1470	156-38-7
24	29.963	0.72	Benzeneacetic acid, 4-hydroxy-	C8H8O3	152	1470	156-38-7
25	30.158	0.72	4-(2-Methoxyethyl)phenol	C9H12O2	152	1289	56718-71-9
26	31.118	0.72	Benzeneacetic acid, 4-hydroxy-	C8H8O3	152	1470	156-38-7
27	30.825	0.71	Benzeneacetic acid, 4-hydroxy-	C8H8O3	152	1470	156-38-7
28	28.689	0.67	Benzeneacetic acid, 4- hydroxy-, methyl ester	C9H10O3	166	1380	42058-59-3
29	30.196	0.63	Benzeneacetic acid, 4-hydroxy-	C8H8O3	152	1470	156-38-7
30	30.963	0.63	Benzeneacetic acid, 4-hydroxy-	C8H8O3	152	1470	156-38-7
31	31.023	0.63	Benzeneacetic acid, 4-hydroxy-	C8H8O3	152	1470	156-38-7
32	30.885	0.62	Benzeneacetic acid, 4-hydroxy-	C8H8O3	152	1470	156-38-7
33	28.755	0.57	Benzeneacetic acid, 4-hydroxy-	C8H8O3	152	1470	156-38-7
34	31.517	0.55	Benzeneacetic acid, 4-hydroxy-	C8H8O3	152	1470	156-38-7
35	30.35	0.54	Benzeneacetic acid, 3-hydroxy-	C8H8O3	152	1470	156-38-7
36	30.735	0.53	Benzeneacetic acid, 4-hydroxy-	C8H8O3	152	1470	156-38-7

### The Protective Effect of Strain QSB-6 on Plant Roots

**FIGURE 6:**
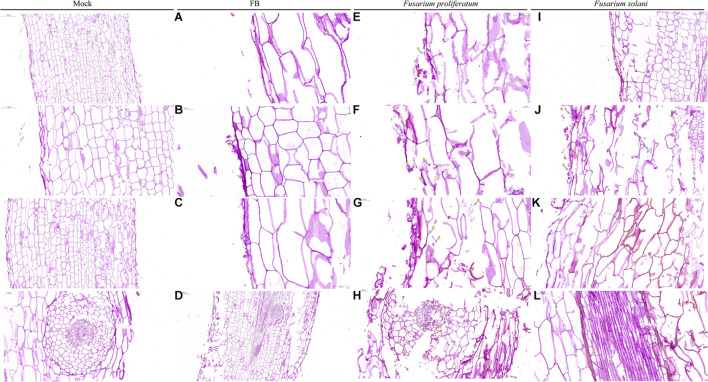
Microscopic observation of the plant root stained by PAS. Mock: sterile distilled water treatment, FB fermentation broth treatment. *Fusarium proliferatum* and *Fusarium solani*: the roots of the plants were soaked with the conidia suspension for 12 h. PAS staining turns the polysaccharides on the fungal wall purple-red. Mock: the root tissue was intact, the cell boundaries are clear and neatly arranged. FB: The conidia and hyphae of *Fusarium* were attached to the epidermis, the epidermis and cortex cells were slightly broken, deformed, and the internal tissue structure is intact **(A–D)**. **(E,G,I)** Dense mycelium appeared in the epidermis of the root system, and the epidermal and cortical cells appeared ruptured, and deformed (arrows), and the cells were arranged irregularly. **(F)** Cauliflower-like structure appeared in the infected root areas (arrows). **(H,J–L)** The conidia and hyphae of *Fusarium* appeared in the cortex and vascular column.

Observation of plant root sections showed that the root system was mainly composed of three parts: (from outside to inside) the epidermis, the cortex (the outer cortex, the cortical parenchyma, the Kjeldahl belt), and the vascular column (the central sheath, phloem, and xylem). Root epidermal cells infected by *Fusarium* were deformed, broken, detached, and/or irregularly arranged. Fungal hyphae invaded the cortical cells through the intercellular layer, then entered the vascular column. New hyphae were visible mainly close to the cell wall, and mature hyphae were scattered in the cells. There was also a large amount of cell contents (viscous substances and starch granules) in the cortical cells and vascular column (Figure 6 and Supplementary Figure 8). The epidermal and cortical cells treated with both strain QSB-6 and *Fusarium* appeared slightly shrunken and ruptured, and the conidia and hyphae of *Fusarium* were attached only to the root epidermal cells (Figures 6A–D). Root systems from the control treatment were complete and neatly arranged (Figure 6, Mock).

### Effects of Different Treatments on the Growth of *M. hupehensis* Rehd. Seedlings

The CK2, T2, and T1 treatments significantly promoted the growth of plants in July, August, and September. The relative treatment effects were ranked from high to low: CK2 > T2 > T1 > CK1 (Table 4). In September, plant height, ground diameter, aboveground fresh weight, belowground fresh weight, aboveground dry weight, and belowground dry weight were 1.66, 1.32, 1.67, 1.39, 2.53, and 2.49 times higher in the T2 treatment that in the T1 treatment.

**TABLE 4:** Effect of different treatments on seedling biomass of *Malus hupehensis* Rehd.

Treatment	Sampling time	Plant height/cm	Ground diameter/mm	Aboveground fresh weight/g	Underground fresh weight/g	Aboveground dry weight/g	Underground dry weight/g
CK1	July	21.75 ± 0.42c	3.77 ± 0.19c	3.84 ± 0.23c	2.62 ± 0.14c	1.39 ± 0.09c	0.69 ± 0.03c
	August	30.10 ± 0.70d	4.18 ± 0.16d	8.60 ± 0.19d	4.81 ± 0.16d	3.41 ± 0.17d	1.77 ± 0.03d
	September	42.20 ± 2.36c	6.37 ± 0.30d	15.48 ± 2.29c	8.75 ± 0.25c	4.66 ± 0.03c	2.61 ± 0.29c
CK2	July	41.94 ± 1.79a	6.08 ± 0.28a	17.34 ± 1.06a	10.62 ± 0.66a	6.28 ± 0.52a	2.37 ± 0.25a
	August	72.17 ± 1.19a	10.44 ± 0.11a	50.29 ± 2.14a	25.09 ± 1.21a	31.57 ± 0.93a	12.27 ± 0.18a
	September	91.57 ± 4.35a	11.63 ± 0.42a	70.59 ± 9.06a	31.98 ± 4.14a	38.89 ± 3.78a	15.56 ± 1.73a
T1	July	25.02 ± 0.82b	4.49 ± 0.15bc	8.57 ± 0.15b	5.16 ± 0.56b	3.01 ± 0.12b	1.42 ± 0.22b
	August	39.33 ± 0.46c	6.29 ± 0.10c	17.41 ± 1.24c	10.43 ± 0.02c	6.53 ± 0.21c	2.28 ± 0.09c
	September	48.87 ± 0.82c	7.61 ± 0.16c	29.87 ± 4.46c	14.85 ± 0.91bc	12.64 ± 0.18c	4.63 ± 0.05c
T2	July	26.71 ± 0.11b	4.61 ± 0.26b	9.00 ± 0.36b	5.41 ± 0.45b	3.36 ± 0.48b	1.88 ± 0.08ab
	August	68.47 ± 0.41b	9.64 ± 0.10b	37.27 ± 1.37b	16.36 ± 0.75b	25.32 ± 0.62b	8.89 ± 0.19b
	September	81.00 ± 0.40b	10.68 ± 0.22b	49.80 ± 1.54b	20.65 ± 0.79b	31.99 ± 1.20b	11.52 ± 0.59b

*Values are mean ± SD.*

*Seedlings, including plant height, ground diameter, aboveground fresh weight, underground fresh weight, aboveground dry weight, and underground dry weight. CK1, 31-year-old orchard soil; CK2, methyl bromide fumigation; T1, fertilizer carrier; T2, QSB-6 bacterial fertilizer. Different letters indicate significantly different at 5% level by Duncan’s new multiple range test. The same below.*

### Effects of Different Treatments on Plant Roots

**FIGURE 7:**
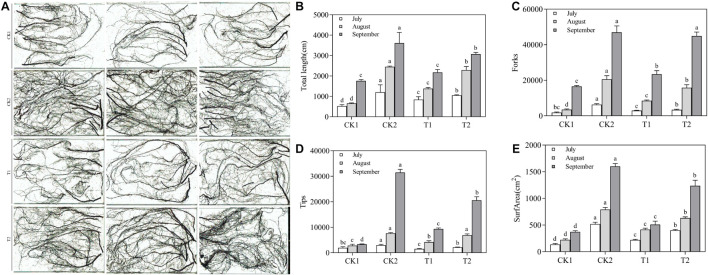
Effect of different treatments on the root architecture of *Malus hupehensis* Rehd. seedlings. **(A)** Root system scan obtained by Microtek ScanMaker i800 Plus, **(B)** total root length, **(C)** the number of root bifurcation, and **(D)** root tips, **(E)** root surface area.

Root structure clearly differed among the different treatments (Figure 7A). In July, there were no significant differences in length, surface area, tips, and forks among the different treatments. In September, the plants had grown considerably, and root length, surface area, tips, and forks were significantly lower in the CK1 treatment than in the CK2 and T2 treatments. The length, surface area, tips, and forks were 1.74, 3.32, 6.08, and 2.17 times higher in the T2 treatment that in the CK1 treatment (Figures 7B–E). The root respiration rate and the SOD, POD, and CAT activities increased from July to August and September in all treatment groups. These increases were significantly higher in the T2 treatment than in CK1, whose values were close to those of CK2 (Table 5). In September, the root respiration rate of T2 was 1067.27 μmol O_2_⋅g^–1^ FW⋅min^–1^, which was 1.53 and 1.36 times higher than those of CK1 and T1. The activities of SOD, POD, and CAT in the T2 treatment were 1.50, 2.87, and 1.74 times higher than those in CK1. MDA content showed the opposite trend: compared with CK1, MDA content of T2 decreased by 37.65, 59.82, and 78.10% in July, August, and September, respectively.

**TABLE 5:** The effects of different treatments on root protective enzyme activity and root vitality.

Treatment	Sampling time	SOD activity (U min^–1^ g^–1^FW)	POD activity (U min^–1^ g^–1^FW)	CAT activity (U min^–1^ g^–1^FW)	MDA activity (μmol g^–1^FW)	Respiration rate of root (μmol O_2_⋅g^–1^FW⋅min^–1^)
CK1	July	105.16 ± 1.84d	4.16 ± 0.05d	11.35 ± 0.01d	8.10 ± 0.09a	138.77 ± 8.38d
	August	155.03 ± 0.94d	6.88 ± 0.02d	28.11 ± 1.36d	11.30 ± 0.23a	427.10 ± 2.83d
	September	192.82 ± 0.78d	10.09 ± 0.36d	35.63 ± 0.89d	15.89 ± 0.06a	695.50 ± 11.48f
CK2	July	231.69 ± 0.78a	15.65 ± 0.20a	33.11 ± 0.07a	3.01 ± 0.12d	423.12 ± 2.24a
	August	285.88 ± 0.43a	20.65 ± 0.09a	53.15 ± 1.42a	3.22 ± 0.19d	819.66 ± 36.97a
	September	311.79 ± 0.22a	32.29 ± 0.08a	71.47 ± 0.47a	2.66 ± 0.19d	1189.52 ± 8.39a
T1	July	152.16 ± 2.04c	7.60 ± 0.06c	18.92 ± 0.14c	6.22 ± 0.05d	199.02 ± 5.77b
	August	180.08 ± 0.99c	10.49 ± 0.33c	32.88 ± 0.54c	5.28 ± 0.22b	549.79 ± 5.32c
	September	231.26 ± 0.75c	17.49 ± 0.07c	45.17 ± 1.08c	5.03 ± 0.02b	787.21 ± 3.39e
T2	July	207.50 ± 3.82b	11.81 ± 0.01b	26.46 ± 0.39b	5.05 ± 0.03c	357.23 ± 3.58b
	August	244.21 ± 0.99b	18.60 ± 0.15b	45.73 ± 0.70b	4.54 ± 0.06c	685.07 ± 3.37b
	September	289.77 ± 0.78b	28.96 ± 0.06b	61.84 ± 0.33b	3.48 ± 0.10c	1067.27 ± 14.48b

*Different letters indicate significantly different at 5 % level by Duncan’s new multiple range test.*

### Effect of Different Treatments on the Soil Microbial Community

**FIGURE 8:**
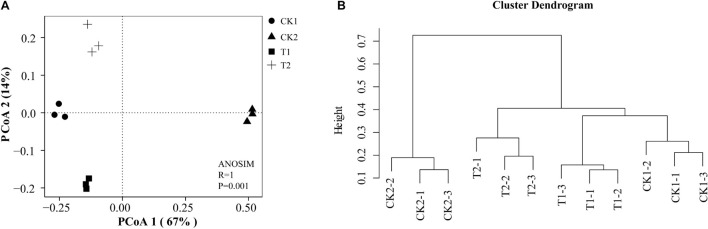
Fungi community structures in the different treatments. Principal coordinate analysis (PCoA) **(A)** and cluster analysis **(B)** plot based on the OTUs of Bray–Curtis distance. *R* = 1 > 0, *p* < 0.05 means that the difference between groups is greater than the difference within groups, and there are significant differences between different treatments. Each treatment contains three repetitions.

The number of soil bacteria increased significantly after T2 treatment in July, August, and September; their numbers were 12.73, 9.72, and 9.64 times higher in T2 than in CK1 (Table 6). In September, the number of soil fungi differed significantly among treatments. Soil fungal numbers were reduced by 85.58, 17.31, and 81.74% in CK2, T1, and T2 compared with CK1. The number of soil fungi after T1 treatment increased by 13.19% in September compared with July. The number of actinomycetes in the soil and the ratio of soil bacteria/fungi were significantly higher in the T2 treatment than in CK1. The soil bacteria/fungi ratios in July, August, and September could be ranked T2 > CK2 > T1 > CK1. The real-time quantitative PCR results indicated there was a significant difference (*p* > 0.05) in CFU levels of *F. proliferatum*, *F. verticillioides*, *F. oxysporum*, and *F. solani* in the soil of different treatments (Table 6). Compared with CK1, the abundance of the four *Fusarium* species were significantly lower in the CK2 and T2 treatments in July, August, and September. In September, The abundance of *F. proliferatum*, *F. verticillioides*, *F. oxysporum*, and *F. solani* decreased by 81.30, 71.08, 59.26, and 50.88% in the T2 treatment compared with CK1, respectively. The results of PCoA (Figure 8A) and cluster analysis (Figure 8B) showed that the fungal community structures of the T2 and CK2 treatments differed significantly from that of CK1, and the fungal community structures of T1 and CK1 were similar. These results showed that strain QSB-6 also had a good inhibitory effect on the growth of *Fusarium* in the soil environment.

**TABLE 6:** The effect of strain QSB-6 on the density of microorganisms in the rhizosphere of *Malus hupehensis* Rehd. seedlings.

Treatment	Sampling time	Bacteria density (×10^5^ CFU/g soil)	Fungi density (×10^3^ CFU/g soil)	Actinomycete density (×10^6^ CFU/g soil)	Bacteria population (×10^5^ CFU/g soil)	Number of four *Fusarium* copys/g soil			
						*Fusarium oxysporum* (×10^10^)	*Fusarium verticillioides* (×10^6^)	*Fusarium solani* (×10^11^)	*Fusarium proliferatum* (×10^6^)
CK1	July	10.00 ± 0.58b	29.00 ± 1.53a	18.67 ± 1.45d	34.86 ± 3.66b	1.06 ± 0.01a	1.58 ± 0.06a	1.96 ± 0.04a	1.89 ± 0.04a
	August	9.33 ± 0.33b	30.33 ± 2.73a	14.67 ± 2.19c	31.24 ± 2.75c	1.07 ± 0.02a	1.65 ± 0.02a	2.09 ± 0.04a	2.10 ± 0.12a
	September	8.33 ± 0.88d	34.67 ± 1.45a	18.00 ± 1.15c	24.16 ± 2.82c	1.08 ± 0.01a	1.66 ± 0.02a	2.26 ± 0.03a	2.30 ± 0.03a
CK2	July	3.33 ± 0.88c	4.67 ± 1.45c	11.33 ± 0.88e	110.95 ± 69.53b	0.40 ± 0.01d	0.44 ± 0.01d	1.03 ± 0.01d	0.51 ± 0.03d
	August	14.67 ± 1.45b	4.00 ± 0.58b	12.67 ± 1.20c	371.67 ± 17.40b	0.40 ± 0.00d	0.44 ± 0.01d	1.05 ± 0.01d	0.47 ± 0.02c
	September	28.33 ± 2.60b	5.00 ± 1.15c	23.67 ± 2.73c	620.00 ± 117.19b	0.40 ± 0.00d	0.44 ± 0.01d	1.03 ± 0.03d	0.47 ± 0.01c
T1	July	12.33 ± 1.20b	25.33 ± 1.45a	39.33 ± 2.33b	48.49 ± 2.57b	0.87 ± 0.01b	0.99 ± 0.00b	1.81 ± 0.02b	1.20 ± 0.03b
	August	15.67 ± 0.88b	28.00 ± 1.15a	46.33 ± 1.86b	56.24 ± 4.67c	0.90 ± 0.01b	0.98 ± 0.02b	1.84 ± 0.02b	1.25 ± 0.02b
	September	17.00 ± 2.08c	28.67 ± 2.03b	61.00 ± 4.73b	60.05 ± 8.51c	0.93 ± 0.01b	1.03 ± 0.01b	1.91 ± 0.01b	1.50 ± 0.08b
T2	July	127.33 ± 2.19a	15.00 ± 1.53b	58.00 ± 2.08a	870.30 ± 106.55a	0.70 ± 0.01c	0.69 ± 0.02c	1.24 ± 0.04c	0.62 ± 0.01c
	August	90.67 ± 4.18a	8.33 ± 0.88b	66.33 ± 4.37a	1102.02 ± 69.25a	0.59 ± 0.00c	0.53 ± 0.01c	1.17 ± 0.02c	0.59 ± 0.00c
	September	80.33 ± 2.19a	6.33 ± 0.88c	72.67 ± 1.45a	1325.56 ± 205.99a	0.44 ± 0.00c	0.48 ± 0.00c	1.11 ± 0.01c	0.43 ± 0.03c

*Different letters indicate significantly different at 5 % level by Duncan’s new multiple range test.*

## Discussion

### The Effect of Strain QSB-6 on the Growth of *Fusarium* Hyphae and Spore Germination

*Fusarium* spp. include a large number of complex fungi and ascomycete teleomorphs; many *Fusarium* species produce toxic secondary metabolites and/or cause serious plant diseases (Nelson et al., 1983). *F. verticillioides* and *F. proliferatum* are harmful to corn due to their production of fumonisin mycotoxins (Marín et al., 2010). Edel-Hermann and Lecomte (2019) found that *Fusarium* spp. – particularly formae speciales of *F. oxysporum* – were important vascular wilt pathogens of many agricultural crops such as ornamental plants and garden crops. *Fusarium* has been identified as a dominant pathogen in rhizosphere soil from continuously cropped soybean and potato fields (Meng et al., 2012; Bai et al., 2015), and also has been reported to be one of the potential soil pathogens that cause ARD (Van Schoor et al., 2009; Kelderer et al., 2012; Wang G. S. et al., 2018). At present, biological control methods have been widely used to control *Fusarium* spp., and their main mechanisms include antagonism, competition, and induction (Weller et al., 2002; Mazurier et al., 2009; Kejela et al., 2016; Gómez Expósito et al., 2017). The measurement of antagonistic activity (i.e., the dual−culture plate assay) is the most commonly used method for screening biocontrol bacteria (Hermosa et al., 2000; Gómez Expósito et al., 2017). Agarwal et al. (2017) used the above method to isolate a *B. pumilus* MSUA3 from higher altitude of Himalayan ranges capable, which can strongly inhibited the growth of *R. solani* and *F. oxysporum* by producing chitinolytic enzymes and an antibiotic surfactin, causing lysis, and deformities in the hyphae. Chang et al. (2007) isolated a strain of *B. cereus* QQ308 that can grow on shellfish chitin wastes, which can secrete a complex of hydrolytic enzymes, including chitinase, chitosanase, and protease, inhibited spore germination and germ tube elongation of *F. oxysporum*, *F. solani*, and *P. ultimum*.

### Analysis of Metabolites From Strain QSB-6

In this study, *B. amyloliquefaciens* strain QSB-6, which showed good inhibitory effects toward a variety of pathogens, was screened by the flat standoff test. *Fusarium* spore germination rate was reduced by more than 60% relative to the control following treatment with fermentation broth and cell-free culture filtrate. Strain QSB-6 also can strongly inhibited the growth of *Fusarium* by producing antibacterial substances, causing lysis and deformities in the hyphae. The above results indicated that strain QSB-6 is a good biological control agent against *Fusarium*. However, Many biocontrol bacterial strains are sensitive to external conditions (i.e., light, high temperature, soil pH extremes), resulting in poor control efficacy in the field (Alabouvette et al., 2009; Armin et al., 2021). We therefore investigated the physical and chemical properties of extracellular metabolites from strain QSB-6. It showed good thermal and acid-base stability and was not sensitive to ultraviolet and visible light, indicating that strain QSB-6 has good development potential and application prospects.

The metabolites produced by microorganisms are very complex, and their specific molecular structures are also very diverse. The antibacterial substances produced by specific strains also differ. Antibacterial substances that inhibit pathogens tend not to act alone, but instead two or more antibacterial substances act synergistically to produce antibacterial effects (Hyakumachi et al., 2014; Carrión et al., 2019; Joo et al., 2020; Newman and Cragg, 2020; Armin et al., 2021). 2,4-Diacetylphloroglucinol (DAPG) and phenazines (PHZ) produced by *Pseudomonas* species have been used as antibiotics to control disease suppressive soils and *Fusarium wilt* (Weller et al., 2002; Haas and Defago, 2005; Mazurier et al., 2009; Raaijmakers and Mazzola, 2012). Kejela et al. (2016) isolated *B. amyloliquefaciens* strain BT42 from the root rhizosphere and demonstrated that it can antagonize *Colletotrichum gloeosporioides* and *F. oxysporum* and promote plant growth. Its main secondary metabolite that inhibits pathogenic fungi is harmine (β-carboline alkaloids). Jeong et al. (2017) found that *B. licheniformis* MH48 and its metabolite, benzoic acid, clearly show antifungal activity against *R. solani* and *C. gloeosporides*. Zhao et al. (2010) isolated an antifungal compound from the *n*-butanol extract of culture filtrate of *B. vallismortis* ZZ185 and identified as Bacillomycin D (n-C14 and iso-C15), which has strong *in vitro* inhibition activity against plant pathogens such as *F. graminearum*, *A. alternata*, and *R. solani*.

In the present experiment, we used test-tube detection and thin-layer chromatography chromogenic methods to characterize the extract phase of fermentation broth from strain QSB-6 and found that its constituent metabolites included steroid, lactones, coumarins and their glycosides, flavonoids, cardiac glycosides, amino acids, peptides and proteins, sugars, polysaccharides and glycosides, phenols, alkaloids, organic acid, coumarins, and saponin. Most are reported to be antibacterial active ingredients in plant extracts, with no occur residual and toxic side effects, Some are used as anti-bacterial antiseptic additives (Demirtas et al., 2013; Djeussi et al., 2013; Desta et al., 2015; Móricz et al., 2015). The toxic components to *Fusaria* were identified by GC–MS as organic acid esters produced by strain QSB-6. Among them, 1,2-benzenedicarboxylic acid and benzeneacetic acid, 3- hydroxy-, methyl ester had the strongest inhibitory effects on *Fusarium* growth. This result provides a theoretical foundation for the development of new microbial fungicides.

### Effect of QSB-6 Fertilizer Treatment on Plant Roots

*Bacillus* can produce antagonistic substances to inhibit the growth of plant pathogens by colonizing the plant rhizosphere or body surface. It may also compete with pathogens for space and/or nutrition or induce plant resistance, thereby achieving the purpose of biological control (Junaid et al., 2013; Jambhulkar et al., 2015; Gómez Expósito et al., 2017). Many studies have shown that plant resistance involves the accumulation of reactive oxygen species, antioxidant enzymes, and lignin (Pshibytko et al., 2006; Dodds and Rathjen, 2010; Li et al., 2016). The plant antioxidant enzyme system includes SOD, POD, CAT, and other enzymes (Sharma and Dubey, 2007; Radwan et al., 2010; Zhang Y. et al., 2016). The enhancement of plant antioxidant enzyme activity can reduce the accumulation of MDA in response to oxidative stress, maintaining the stability of the cell membrane and enhancing plant tolerance (Balal et al., 2012). At the same time, *Bacillus* can also promote plant growth by degrading organic matter to release plant nutrients, improve root vitality, and optimize root architecture (Glick, 1995; Nagórska et al., 2007). Root morphological parameters play an important role in plant development because nutrient uptake and water absorption capacity is more dependent on root length and root surface area than on total root biomass (Boot, 1989; Bresson et al., 2013; Syed-Ab-Rahman et al., 2019). The results of this study are consistent with these proposed mechanisms. QSB-6 fertilizer treatment (T2) promoted significant increases in plant root respiration rate and protective enzyme activities (SOD, POD, and CAT); MDA content was reduced, and root length, surface area, tips, and forks were significantly increased, thereby promoting greater plant growth. The promotion of root growth may also be related to the metabolites produced by strain QSB-6. The length of primary roots and the number of lateral roots of *A. thaliana* plantlet treated with metabolites (1,2-benzenedicarboxylic acid and benzeneacetic acid, 3- hydroxy-, methyl ester) increased significantly. Plant roots treated with strain QSB-6 also showed less damage from *Fusarium*, suggesting that QSB-6 had colonized the host plant surface or the infection site, forming a biofilm on the root epidermis and preventing pathogen intrusion (Raupach and Kloepper, 1998; Romero et al., 2004; Ji et al., 2008), consistent with the results of Chen et al. (2012). Large amounts of viscous substances and starch granules were also produced in the infected root cortical cells and vascular cylinders. Viscous substances present a strong mechanical barrier that inhibits microbial invasion and can also prevent the longitudinal expansion of microbial infection by blocking the xylem vessel (Daayf et al., 1995; Lagopodi et al., 2002). *Fusarium* can be classified morphologically as Nectria-like. Intracellular condensed nodular fungal structures with the shape of a cauliflower head are reported to occur in infected root areas (Grunewaldt-Stöcker et al., 2020), and similar structures were observed in this experiment.

### The Effect of QSB-6 Fertilizer Treatment on the Soil Microbial Community

In recent years, *Bacillus* species have been considered as promising agents for the control of diverse plant pathogens. *Bacillus* are applied primarily in the form of a solid or liquid fertilizers (Shahena et al., 2021), and they have shown a good control effect in cucumber (*Fusarium wilt*, downy mildew, etc.), pepper (bacterial wilt, soft rot, etc.), rice (rice blast, sheath blight, etc.), wheat (root rot, leaf blight, etc.), and other crop diseases (Baker et al., 1983; Wilson and Pusey, 1985; Lin et al., 2001; Chan et al., 2003; Souto et al., 2004; Ouhaibi-Ben Abdeljalil et al., 2016; Zalila-Kolsi et al., 2016; Zhang M. et al., 2016; Fan et al., 2017b; Gómez Expósito et al., 2017; Qin et al., 2017; Barratt et al., 2018; Wang X. Q. et al., 2018). Ramírez et al. (2021) isolated a *B. cereus* MH778713 from root nodules of *Prosopis laevigata*, and the volatile hentriacontane and 2,4-di-tert-butylphenol produced by it can effectively inhibited the radial growth of *F. oxysporum* and protected tomato plants against *Fusarium wilt*. Huang et al. (2012) developed a novel bio-organic fertilizer (BIO) by fermenting mature compost with *B. pumilus* N43 can effectively control of *Rhizoctonia solani* damping-off disease in cucumber. The QSB-6 bacterial fertilizer used in this experiment adopts solid state fermentation (cow manure: straw = 3:1), which has the advantages of inexpensive substrates, continuality of the process, low waste volumes, and beneficial to the survival of microorganisms (Wang et al., 2009; Dubey and Dubey, 2010; Duan et al., 2020). In September, the effects of QSB-6 bacterial fertilizer and methyl bromide fumigation treatment (Mbr) were almost as good, and both significantly promoted the growth of plant seedlings. When applying in the field, band placement (planting tree row) and localized placement (tree hole) is used and the application rate is about 240-300 g/m^2^ (400–460 USD/acre). Compared with the common fumigants dimethyl disulfide (DMDS) (60 g/m^2^), Methyl bromide (568.50 USD/acre), and 1,3-dichloropropene (600 USD/acre) in production, the cost is lower and it is more conducive to farmers’ income (Raski et al., 1976; Pecchia et al., 2017). The above results indicated that strain QSB-6 has the potential to become an excellent biological control agent to replace fumigants to control ARD.

Previous studies have shown that the occurrence of ARD is closely related to changes in the composition of the microbial community in orchard soils. Long-term replant cropping will lead to an imbalance in the rhizosphere microbial population, reducing beneficial microbes and increasing the abundance of soil-borne fungal pathogens, ultimately leading to a decrease in crop yield (Mazzola, 1998; Yim et al., 2013; Franke-Whittle et al., 2015; Spath et al., 2015). At present, T-RFLP, qPCR, and DNA stable isotope probing (DNA-SIP) techniques are often used to study soil microbial community structure (Cretoiu et al., 2013; Gómez Expósito et al., 2017; Moein et al., 2019). Shennan et al. (2018) found that when rice bran was utilized as the ASD carbon input, significant changes in bacterial and fungal community composition were observed in the soil as characterized by T-RFLP analysis. Here, we used T-RFLP and qPCR to show that QSB-6 fertilizer treatment can significantly change the soil fungal community structure, reduce the number of soil fungi, and reduce the abundance of *Fusarium*. A similar finding was reported in a biocontrol study of Watermelon *Fusarium wilt* (Zhao et al., 2014). The above results showed that adding QSB-6 bacterial fertilizer to the soil had a better inhibitory effect on the fungi in the soil, especially *Fusarium*, and the effect was almost as good as the Mbr treatment with the extension of time. The number of bacteria in the soil has also increased significantly, and the soil essentially became a high-fertilizer “bacterial” soil. Strain QSB-6 may consume large amounts of soil nutrients, increase its own growth rate, and thereby reduce the level of nutrients needed for the survival of plant pathogens, inhibiting their growth (Gómez Expósito et al., 2017; Shi et al., 2020). It shows that the application of QSB-6 bacterial fertilizer can provide a healthy soil microbial environment for the growth of plant seedlings. Strain QSB-6 is classified as first level (i.e., exempt from toxicological testing) according to the general technical guidelines for microbial fertilizer safety. This safe and non-pathogenic microorganism can be used as a green, environmentally friendly biocontrol agent for ARD.

## Data Availability Statement

The datasets presented in this study can be found in online repositories. The names of the repository/repositories and accession number(s) can be found in the article/Supplementary Material.

## Author Contributions

ZM and CY contributed to conception and design of the study. YD organized the database. YD, RC, RZ, and WJ performed the statistical analysis. YD wrote the first draft of the manuscript and contributed in the GC–MS analysis. RC and RZ wrote sections of the manuscript. All authors contributed to manuscript revision, read, and approved the submitted version.

## Conflict of Interest

The authors declare that the research was conducted in the absence of any commercial or financial relationships that could be construed as a potential conflict of interest.

## Publisher’s Note

All claims expressed in this article are solely those of the authors and do not necessarily represent those of their affiliated organizations, or those of the publisher, the editors and the reviewers. Any product that may be evaluated in this article, or claim that may be made by its manufacturer, is not guaranteed or endorsed by the publisher.

## Footnotes

1
https://www.ncbi.nlm.nih.gov/

2
http://www.phylo.org
