# Supplementary material for: Isolation, Identification, and Antibacterial Mechanisms of Bacillus amyloliquefaciens QSB-6 and Its Effect on Plant Roots
Source: Front Microbiol. 2021 Sep 16;12:746799. doi: 10.3389/fmicb.2021.746799 (PMC8482014; doi:10.3389/fmicb.2021.746799)
Supplement: Supplementary file 1 [file Data_Sheet_1.docx]

**Isolation, identification, and antibacterial mechanisms of *Bacillus amyloliquefaciens* QSB-6 and its effect on plant roots**

Yanan Duan ^1^, Ran Chen^1^, Rong Zhang^1^, Weitao Jiang^1^, Xuesen Chen^1^, Chengmiao Yin^1*^, and Zhiquan Mao^1*^

^1^National Key Laboratory of Crop Biology, College of Horticulture Science and Engineering, Shandong Agricultural University, Shandong 271018, PR China

***Correspondence:**

Zhiquan Mao, Email: [mzhiquan@sdau.edu.cn](mailto:mzhiquan@sdau.edu.cn)

Chengmiao Yin, E-mail: [yinchengmiao@163.com](mailto:yinchengmiao@163.com)

**Supplementary information**

**Table S1:** Sample location, separation of bacteria and biocontrol bacteria in different parts of the plant and rhizosphere soil, and The total number of biocontrol bacteria exhibited effective inhibitory activity against on *Fusarium proliferatum*, *Fusarium verticillioides*, *Fusarium oxysporum*, *Fusarium solani*, *Alternaria alternata*, *Phoma* sp., *Aspergillus flavus*, *Rhizoctonia solani*, *Penicillium brasilianum*, *Albifimbria verrucaria*, and *Valsa mali*, respectively.

| **Province** | | **Shandong** | | | |
| --- | --- | --- | --- | --- | --- |
| **Local site** | | **Sujiadian Town, Qixia City** | **Shewobo Town, Qixia City** | **Lutou Town, Longkou City** | **Yulindian Town, Muping District** |
| Location numbera^1^ | | Q | S | L | M |
| Latitude and longitude | | Lon:120.68587  Lat:37.424576 | Lon:120.870898  Lat:37.132936 | Lon:120.465274  Lat:37.606707 | Lon:121.629079  Lat:37.278035 |
| Number of bacteria | Soil | 44 | 38 | 83 | 40 |
|  | Root | 14 | 6 | 12 | 23 |
|  | Stem | 8 | 23 | 36 | 28 |
|  | Leaf | 27 | 27 | 28 | 44 |
|  | Fruit | 0 | 17 | 11 | 0 |
|  | Total | 93 | 111 | 170 | 135 |
| Number of biocontrol bacteria | Soil | 24 | 28 | 46 | 15 |
|  | Root | 11 | 0 | 1 | 11 |
|  | Stem | 8 | 11 | 30 | 9 |
|  | Leaf | 26 | 4 | 5 | 12 |
|  | Fruit | 0 | 8 | 7 | 0 |
|  | Total | 69 | 51 | 89 | 47 |
| *Fusarium proliferatum* | | 26 | 14 | 36 | 24 |
| *Fusarium verticillioides* | | 25 | 12 | 42 | 35 |
| *Fusarium oxysporum* | | 20 | 11 | 24 | 28 |
| *Fusarium solani* | | 26 | 14 | 36 | 20 |
| *Alternaria alternata* | | 25 | 18 | 39 | 19 |
| *Aspergillus flavus* | | 6 | 5 | 10 | 9 |
| *Phoma* sp. | | 7 | 13 | 35 | 16 |
| *Valsa mali* | | 9 | 18 | 33 | 22 |
| *Rhizoctonia solani* | | 14 | 8 | 31 | 6 |
| *Penicillium brasilianum* | | 6 | 6 | 12 | 4 |
| *Albifimbria verrucaria* | | 5 | 2 | 8 | 3 |

^1^Naming rules: place name abbreviation + habitat (soil, root, stem, leaf, fruit) + microorganism type + number. For example, the first strain of bacteria screened in the soil of Sujiadian Town in Qixia was named QSB-1.

**Table S2:** Oligonucleotide primers and probes used used in this experiment.

| Primer name | Oligonucleotide sequence (5′–3′) | Gene | Tm°C | Reference |
| --- | --- | --- | --- | --- |
| 27F/1492R | AGAGTTTGATCCTGGCTCAG-3  GGTTACCTTGTTACGACTT | 16S rDNA | 55℃ | Somerville et al., 2020 |
| 42f/1066r | CAGTCAGGAAATGCGTACGTCCTT  CAAGGTAATGCTCCAGGCATTGCT | gyrA | 62℃ | Chun and Bae 2000 |
| up1f/up2r | GAAGTCATCATGACCGTTCTGCAYGCNGGNGGNAARTTYGA  AGCAGGGTACGGATGTGCGAGCCRTCNACRTCNGCRTCNGTCAT | gyrB | 60℃ | Yamamoto and Harayama 1995 |
| Prpo1/Prpo2 | A TTTCGTT AGCCGAAGAACGT  A TGTGTCCT A TTGAGACACCA | rpoB | 50℃ | Zalila-Kolsi et al., 2016 |
| JR/JF | CATACCACTTGTTGTCTCGGC  GAACGCGAATTAACGCGAGTC | *Fusarium oxysporum* | 60℃ | - |
| CHR/CHF | GACTCGCGAGTCAAATCGCGT  GGGGTTTAACGGCGTGGCC | *Fusarium verticillioides* | 60℃ | - |
| CR/CF | GATCGGCGAGCCCTTGCGGCAAG  CGCCGCGTACCAGTTGCGAGGGT | *Fusarium proliferatum* | 65℃ | - |
| FR/FF | CGAGTTATACAACTCATCAACC  GGCCTGAGGGTTGTAATG | *Fusarium solani* | 65℃ | - |
| ITS1F-FAM/ITS4R | CTTGGTCATTTAGAGGAAGTAA  TCCTCCGCTTATTGATAGC | ITS | 50℃ | Gardes and Bruns 1993 |

**Table S3:** The results of vitro antibacterial activity of crude extract.

| Extraction solvent | Crude extract/g | Inhibition zone /cm | | | | | | | | |
| --- | --- | --- | --- | --- | --- | --- | --- | --- | --- | --- |
|  |  | *Aspergillus flavus* | *Rhizoctonia solani* | *F. solani* | *F. oxysporum* | *F. proliferatum* | *Valsa mali* | *Phoma* sp. | *Alternaria alternata* | 1. *verticillioides* |
| N-butanol | 3.1879 | 0 | 0.1 | 0.3 | 0.2 | 0.1 | 0.1 | 0 | 0.1 | 0.2 |
| Ethyl acetate | 1.2591 | 0.5 | 0.2 | 0.4 | 0.3 | 0.7 | 0.5 | 0 | 0.3 | 0.4 |
| Chloroform | 3.8970 | 0.4 | 0.1 | 0.3 | 0.2 | 0.4 | 0.2 | 0.2 | 0.2 | 0.2 |
| Petroleum ether | 0.7366 | 0.3 | 0.1 | 0.1 | 0.1 | 0.2 | 0.1 | 0 | 0.1 | 0.2 |

**Table S4:** The result of antibacterial activity of each component.

| Component | Weight/g | Inhibition zone /cm | | | | | | | |
| --- | --- | --- | --- | --- | --- | --- | --- | --- | --- |
|  |  | *Aspergillus flavus* | *Penicillium brasilianum* | *Albifimbria verrucaria* | *F. solani* | *F. oxysporum* | *F. proliferatum* | *Alternaria alternata* | *F.verticillioides* |
| I | 5.86 | 0 | 0 | 0 | 0 | 0 | 0 | 0 | 0 |
| II | 0.83 | 0.2 | 0.2 | 0 | 0 | 0.1 | 0.1 | 0 | 0.2 |
| Ⅲ | 0.68 | 0 | 0 | 0 | 0 | 0 | 0 | 0 | 0 |
| Ⅳ | 0.06 | 0.3 | 0.2 | 0 | 0.1 | 0.6 | 0.1 | 0.4 | 0.5 |
| Ⅴ | 4.38 | 0.7 | 0.6 | 0 | 0.3 | 0.6 | 0.6 | 0.5 | 0.5 |
| Ⅵ | 1.26 | 0.2 | 0.5 | 0 | 0 | 0.5 | 0.2 | 0.5 | 0 |

**Table S5:** The basic information of the pure product.

| Name | Purity | Price | Configure concentration | Prostitution |
| --- | --- | --- | --- | --- |
| Terephthalic acid diisopropyl ester | 97% | **342.00/** 100mg | **1 mg/L** | Shanghai Bide Pharmaceutical Technology Co., Ltd. |
| 1,2-Benzenedicarboxylic acid | 98% | 98.00/20mg | **1 mg/L** | Shanghai Jizhi Biochemical Technology Co., Ltd. |
| Carbamic acid, (4-aminophenyl)-, methyl ester | 98% | 1260/1000mg | **1 mg/L** | Shanghai Lin Li Pharmaceutical Technology Co., Ltd. |
| Benzeneacetic acid, 3-hydroxy-, methyl ester | 98% | 120.00/250mg | **1 mg/L** | Shanghai Yuanye Biological Technology Co., Ltd. |

**Table S6:** The physical and chemical properties of the soil in the old apple orchard.

| Location | Nitrate nitrogen  (mg kg^-1^) | Ammonium nitrogen  (mg kg^-1^) | Available Phosphorus  (mg kg^-1^) | Available Potassium(mg kg^-1^) | Organic matter（%） | Soil pH | Soil moisture content（%） | Soil texture |
| --- | --- | --- | --- | --- | --- | --- | --- | --- |
| Manzhuang  Town | 6.18±0.04 | 1.68±0.02 | 10.57±0.37 | 53.13±6.33 | 1.35±0.02 | 6.00±0.23 | 15.49±0.42 | Sandy loam |

**Table S7 :** Utilization ability of strain QSB-6 on 94 phenotypic tests. The Biolog GEN III MicroPlate analyzes a microorganism in 94 phenotypic tests: 71 carbon source utilization assays (columns 1-9) and 23 chemical sensitivity assays (columns 10-12). All of the wells start out colorless when inoculated. During incubation there is increased respiration in the wells where cells can utilize a carbon source and/or grow. Increased respiration causes reduction of the tetrazolium redox dye, forming a purple color. Negative wells remain colorless, as does the negative control well (A-1) with no carbon source. There is also a positive control well (A-10) used as a reference for the chemical sensitivity assays in columns 10-12. All wells visually resembling the A-1(A-10) well should be scored as “negative” (-) and all wells with a noticeable purple color (greater than well A-1 and A-10) should be scored as “positive” (+). Wells with extremely faint color, or with small purple flecks or clumps are best scored as “borderline” (-/+).

| Columns | Nutrient matrix | Reaction type | Columns | Nutrient matrix | Reaction type | Columns | Nutrient matrix | Reaction type |
| --- | --- | --- | --- | --- | --- | --- | --- | --- |
| A1 | Negative Control | _ | C9 | Inosine | -/+ | F5 | D-Glucuronic Acid | -/+ |
| A2 | Dextrin | -/+ | C10 | 1%Sodium Lactate | + | F6 | Glucuronamide | -/+ |
| A3 | D-Maltose | -/+ | C11 | Fusidic Acid | _ | F7 | Mucic Acid | _ |
| A4 | D-Trehalose | + | C12 | D-Serine | + | F8 | Quinic acid | -/+ |
| A5 | D-Cellobiose | + | D1 | D-Sorbitol | + | F9 | D-Saccharic Acid | _ |
| A6 | Gentiobiose | + | D2 | D-Mannitol | + | F10 | Vancomycin | _ |
| A7 | Sucrose | + | D3 | D-Arabitol | _ | F11 | Tetrazolium Violet | -/+ |
| A8 | Turanose | -/+ | D4 | Myo-Inositol | -/+ | F12 | Tetrazolium Blue | _ |
| A9 | Stachyose | -/+ | D5 | Glycerol | + | G1 | p-Hydroxy  -phenylacetic Acid | _ |
| A10 | Positive Control | + | D6 | Glycerol | _ | G2 | Methyl pyruvate | -/+ |
| A11 | pH6 | + | D7 | D- Fructose -6-Phosphate | -/+ | G3 | D-Lactic Acid Methyl Ester | _ |
| A12 | pH5 | + | D8 | D-Aspartic Acid | + | G4 | L-Lactic Acid | + |
| B1 | D-Raffinose | -/+ | D9 | D-Serine | _ | G5 | Citric Acid | + |
| B2 | α-D-Lactose | + | D10 | Troleandomycin | _ | G6 | α-Keto-glutaric Acid | -/+ |
| B3 | D-Melibiose | -/+ | D11 | Rifamycin SV | -/+ | G7 | D-Malic Acid | _ |
| B4 | β-Methyl-D-Glucoside | + | D12 | Minocycline | -/+ | G8 | L-Malic Acid | + |
| B5 | Salicin | + | E1 | Gelatin | + | G9 | Bromosuccinic Acid | -/+ |
| B6 | N-Acetyl-D-Glucosamine | + | E2 | Glycyl-L-Proline | -/+ | G10 | Nalidixic acid | _ |
| B7 | N-Acetyl-β-D-Mannosamine | -/+ | E3 | D-Alanine | + | G11 | Lithium Chloride | + |
| B8 | N-Acetyl-D-Galactosamine | _ | E4 | L-Arginine | -/+ | G12 | Potassium Tellurite | + |
| B9 | N-AcetylNeuraminic acid | _ | E5 | L-Aspartic Acid | + | H1 | Tween 40 | _ |
| B10 | 1% NaCl | + | E6 | L-Glutamic Acid | + | H2 | γ-Amino-Butyric Acid | -/+ |
| B11 | 4% NaCl | + | E7 | L-Histidine | -/+ | H3 | α-Hydroxy-Butyric Acid | － |
| B12 | 8% NaCl | + | E8 | L-Pyroglutamic Acid | -/+ | H4 | β-Hydroxy-D,L-butyric Acid | _ |
| C1 | α-D-Glucose | + | E9 | L-Serine | -/+ | H5 | α-Keto-Butyric Acid | _ |
| C2 | D-Mannose | + | E10 | Lincomycin | _ | H6 | Acetoacetic Acid | _ |
| C3 | D-Fructose | + | E11 | Guanidine HCl | + | H7 | Propionic Acid | _ |
| C4 | D-Galactose | -/+ | E12 | Niaproof 4 | _ | H8 | Acetic Acid | _ |
| C5 | 3-Methyl-D-Glucose | _ | F1 | Pectin | -/+ | H9 | Formic Acid | -/+ |
| C6 | L-Fucose | _ | F2 | Galacturonic acid | -/+ | H10 | Aztreonam | + |
| C7 | D-Fucose | _ | F3 | D-Galactonic Acid Lactone | -/+ | H11 | Sodium Butyrate | + |
| C8 | L-Rhamnose | _ | F4 | D-Gluconic Acid | + | H12 | Sodium Bromate | + |

**Table S8:** Physiological and biochemical characteristics of QSB-6.

| Text index | Results | Text index | Results |
| --- | --- | --- | --- |
| Hydrogen peroxide reaction | + | Arginine bihydrolysis reaction | + |
| Contact enzyme | + | Sucrose fermentation reaction | + |
| Starch hydrolysis enzyme | + | Glucose fermentation reaction | + |
| Nitrate reduction enzyme | + | Methyl red reaction | + |
| Indole enzyme | ^_^ | Voges-Proskauer reaction | + |
| Citrate enzyme | + | Urea enzyme reaction | ^_^ |
| Hydrogen sulfide reaction | + | Gelatin hydrolysis enzyme | + |

Note: +, positive reaction; ^_^, negative reaction. Mean of three replications.

**Table S9:** The Vitro Test of The four Extractions From The Fermentation Fluid of strain QSB-6.

| Test Indigents | Chemical Reactions | Results | Extraction phase | | | |
| --- | --- | --- | --- | --- | --- | --- |
|  |  |  | Ethyl acetate | N-butanol | Chloroform | Petroleum ether |
| Amino acids, peptides and proteins | Biuret reaction | Blue or purple precipitation | + | + | + | - |
|  | 5% sulfuric acid precipitation reaction | First turbid and then precipitate | + | + | + | - |
| Sugars, polysaccharides and glycosides | Basic copper sulfate reaction | Brick red precipitation | - | - | + | + |
|  | alpha-naphthol test | Purple ring is generated | - | - | + | + |
| Phenols | Ferric chloride reaction | The solution is green | + | - | + | - |
|  | Vanillin-hydrochloric acid reaction | Light red | + | - | + | - |
| Alkaloids | Potassium Bismuth Iodide | Orange precipitation | + | - | + | + |
|  | Potassium iodide iodide | Precipitation | + | - | + | + |
| Saponin | Chloroform-concentrated sulfuric acid test | The upper layer is cyan, the lower layer has green fluorescence | + | + | + | + |
|  | Foam test | A lot of bubbles are formed and do not disappear for a long time | + | + | + | + |
| Anthraquinone compounds | Alkaline experiment | Add lye to turn red, acid red disappear | - | - | - | - |
|  | Magnesium acetate experiment | Produces a red reaction | - | - | - | - |
| Flavonoids | Magnesium Hydrochloride Reaction | Red to deep purple | + | - | - | - |
|  | Lead acetate precipitation reaction | A yellow precipitate is formed | + | - | - | - |
| Lactones, coumarins and their glycosides | Iron hydroxamate test | Brown-red or purple reaction | + | + | + | + |
|  | Lactone open-loop and closed-loop test | Turbid-clear-turbid | + | + | + | + |
| Cardiac glycosides | Sodium ferricyanide nitrite test | Red change | + | - | + | - |
|  | Ferric chloride-glacial acetic acid test | The interface is brown | + | - | + | - |
| Steroid | Chloroform-concentrated sulfuric acid test | The upper layer is cyan, the lower layer has green fluorescence | + | + | + | + |
|  | Acetic anhydride-concentrated sulfuric acid test | The solution quickly turned dark green | + | + | + | + |
| Organic acid | pH test paper method | 5-6 | + | + | + | + |
|  | Bromophenol blue ethanol solution | Yellow spots on blue background | + | + | + | + |

Note: +, positive reaction; ^_^, negative reaction. Mean of three replications.

| The Pretesting Component | Streak reagents | Developing agent | Results | Extraction phase | | | |
| --- | --- | --- | --- | --- | --- | --- | --- |
|  |  |  |  | Ethyl acetate | N-butanol | Chloroform | Petroleum ether |
| Steroid | 0.5% phosphomolybdic acid ethanol solution | Chloroform: Acetone=8:2 | Visibly blue purple spots | + | + | + | + |
| Flavonoids | 3% ferric chloride ethanol solution | Acetic acid: water=15:85 | Green, blue and brown spots appear | + | - | - | - |
| Amino acids, peptides and proteins | Ninhydrin solution | N-butanol: acetic acid: water=4:1:5 | Violet spots | + | + | + | - |
| Alkaloids | Bismuth Potassium Iodide Reagent | Chloroform: Acetone=1:1(Ammonia fumigation) | Orange red spots | + | - | + | + |
| Anthraquinone compounds | 10% KOH solution | Petroleum ether: ethyl acetate=8:2 | Fuchsia spots | - | - | - | - |
| Coumarins | Iron Hydroxamic Acid Reagent | N-butanol: acetic acid: water=4:1:1 | Visibly blue purple spots | + | + | + | + |
| Phenols | FeCl_3_, K_3_Fe_3_(CN)_6_ reagent | Chloroform: Acetone=8:2 | Visibly blue-purple spots | + | - | + | - |

**Table S10:** The TLC detection of the four extractions from fermentation fluid of strain QSB-6. Spot the concentrated liquid of each extract phase of strain QSB-6 on a thin-layer chromatography plate, use petroleum ether, chloroform, ethyl acetate, methanol, and acetone in a certain proportion as a developing agent, and spray a variety of streak reagents for testing.

Note: +, positive reaction; ^_^, negative reaction. Mean of three replications.

**Table S11:** The inhibitory effect of 1,2-Benzenedicarboxylic acid on plant fungal pathogens. Different letters indicate significantly different at 5 % level by Duncan’s new multiple range test. Values are mean ± SD.

| Treatment | Concentration(μg/L) | Inhibition rate (%) | Treatment | Concentration(μg/L) | Inhibition rate (%) |
| --- | --- | --- | --- | --- | --- |
| *Fusarium proliferatum* | 10 | 27.74±0.04d | *Alternaria alternata* | 10 | 53.26±0.04d |
|  | 100 | 42.22±0.00c |  | 100 | 55.56±0.00c |
|  | 500 | 52.19±0.04b |  | 500 | 62.15±0.04b |
|  | 1000 | 77.74±0.04a |  | 1000 | 64.44±0.00a |
| *Fusarium verticillioides* | 10 | 27.40±0.37d | *Albifimbria verrucaria* | 10 | 56.85±0.04c |
|  | 100 | 38.78±0.06c |  | 100 | 56.94±0.00c |
|  | 500 | 49.96±0.04b |  | 500 | 63.84±0.05b |
|  | 1000 | 65.52±0.04a |  | 1000 | 76.34±0.05a |
| *Fusarium oxysporum* | 10 | 16.63±0.04d | *Aspergillus flavus* | 10 | 21.07±0.04d |
|  | 100 | 22.15±0.04c |  | 100 | 26.60±0.04c |
|  | 500 | 55.52±0.04b |  | 500 | 43.29±0.04b |
|  | 1000 | 68.85±0.04a |  | 1000 | 77.74±0.04a |
| *Fusarium solani* | 10 | 27.74±0.04d | *Phytophthora* | 10 | 29.96±0.04d |
|  | 100 | 37.70±0.04c |  | 100 | 55.19±0.37c |
|  | 500 | 42.19±0.04b |  | 500 | 74.40±0.04b |
|  | 1000 | 47.61±0.04a |  | 1000 | 87.78±0.00a |
| *Rhizoctonia solani* | 10 | 38.89±0.00d | *Phoma sp.* | 10 | 39.94±0.06d |
|  | 100 | 46.63±0.04c |  | 100 | 46.66±0.61c |
|  | 500 | 55.52±0.04b |  | 500 | 54.47±0.06b |
|  | 1000 | 74.44±0.00a |  | 1000 | 59.94±0.06a |
