# Supplementary material for: Isolation, Identification, and Antibacterial Mechanisms of Bacillus amyloliquefaciens QSB-6 and Its Effect on Plant Roots
Source: Front Microbiol. 2021 Sep 16;12:746799. doi: 10.3389/fmicb.2021.746799 (PMC8482014; doi:10.3389/fmicb.2021.746799)

**Isolation, identification, and antibacterial mechanisms of *Bacillus amyloliquefaciens* QSB-6 and its effect on plant roots**

Yanan Duan ^1^, Ran Chen^1^, Rong Zhang^1^, Weitao Jiang^1^, Xuesen Chen^1^, Chengmiao Yin^1*^, and Zhiquan Mao^1*^

^1^National Key Laboratory of Crop Biology, College of Horticulture Science and Engineering, Shandong Agricultural University, Shandong 271018, PR China

***Correspondence:**

Zhiquan Mao, Email: [mzhiquan@sdau.edu.cn](mailto:mzhiquan@sdau.edu.cn)

Chengmiao Yin, E-mail: [yinchengmiao@163.com](mailto:yinchengmiao@163.com)

**Supplementary information**

**Figure S1:** The isolated strain QSB-6 was cultured on LB agar at 37 ℃ for 24 h (A) and the cellular morphological character were observed by Nikon fluorescence microscope BX-51 (B-C) and scanning electron microscope SU-8010 (D-I).

**
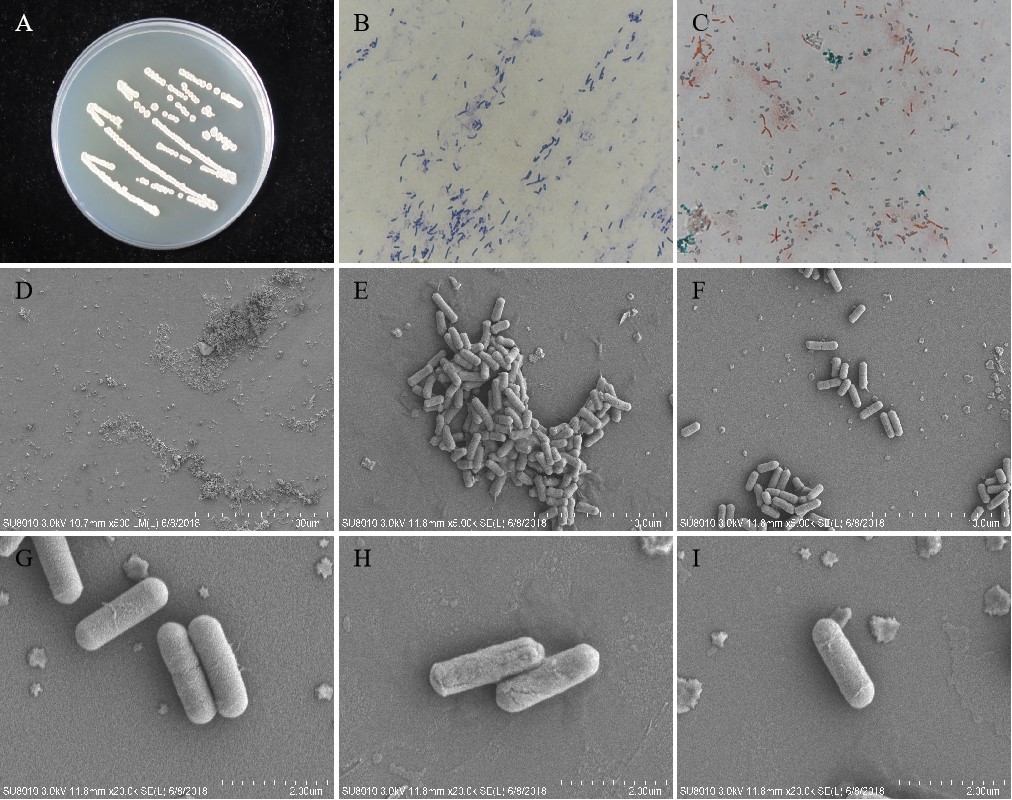
**

Note: A Single colony morphology; B-C The shape and size of the bacteria and spores, bar=100×/1.30 oil; D-I The shape and size of the bacteria, D bar=10.7 mm×500 LM (L), 100μm, E-F bar=11.8 mm×5.00 k SE (L), 10 μm, G-I bar=11.8 mm×20.0 k SE (L), 2 μm.

**Figure S2:** Effect of cell-free culture filtrate on mycelia of *Fusarium solani* (a) and *Fusarium oxysporum* (b) A-B was the blank control mycelium, (a) C-F and (b) C-H was the mycelium treated with cell-free culture filtrate.


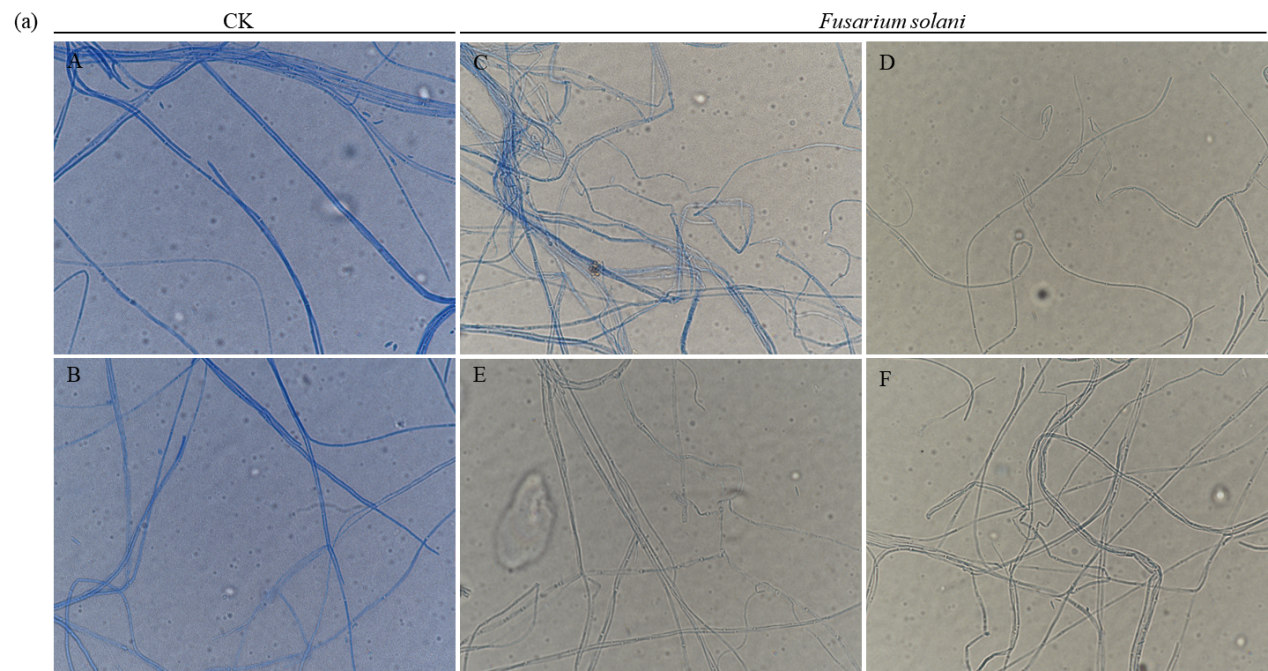

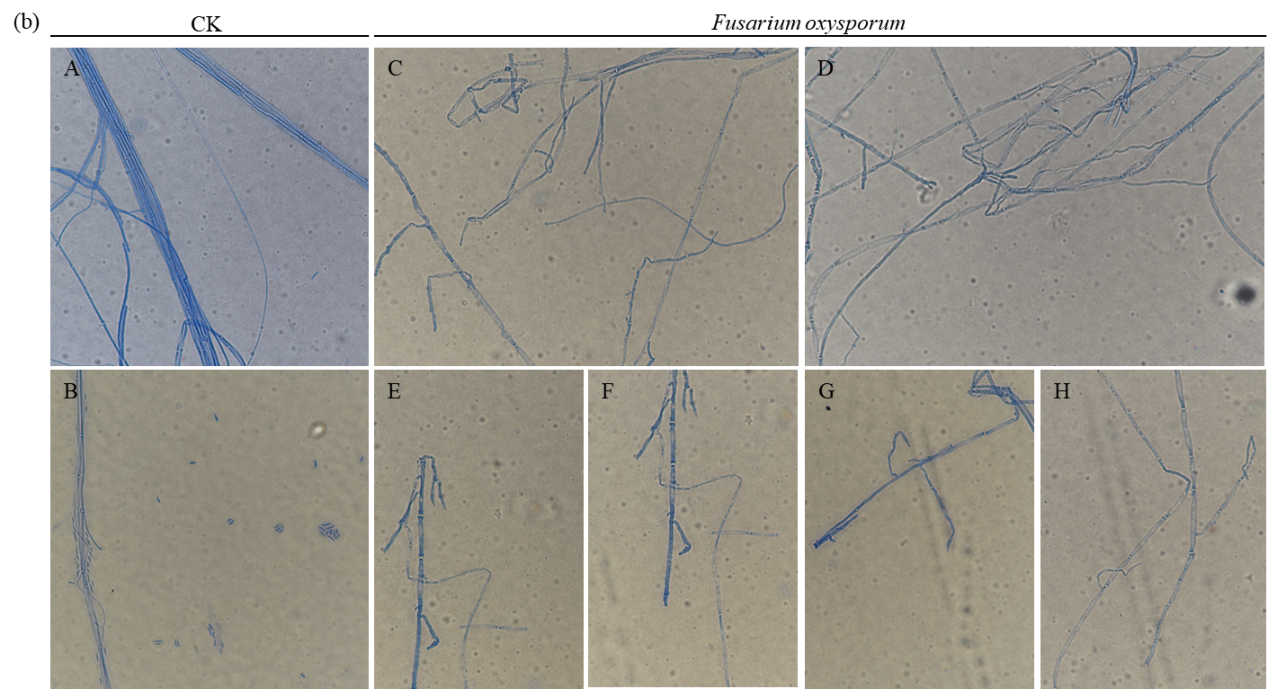


Note: A-B Normal mycelium, bar=20×, C-D Mycelium was irregularly reticulated and swollen, uneven in thickness, (b) E-H The hyphae appear to be curved tip, twisted, thinned, broken, shrunken, shriveled, and the hyphae were dissolved and deformed.

**
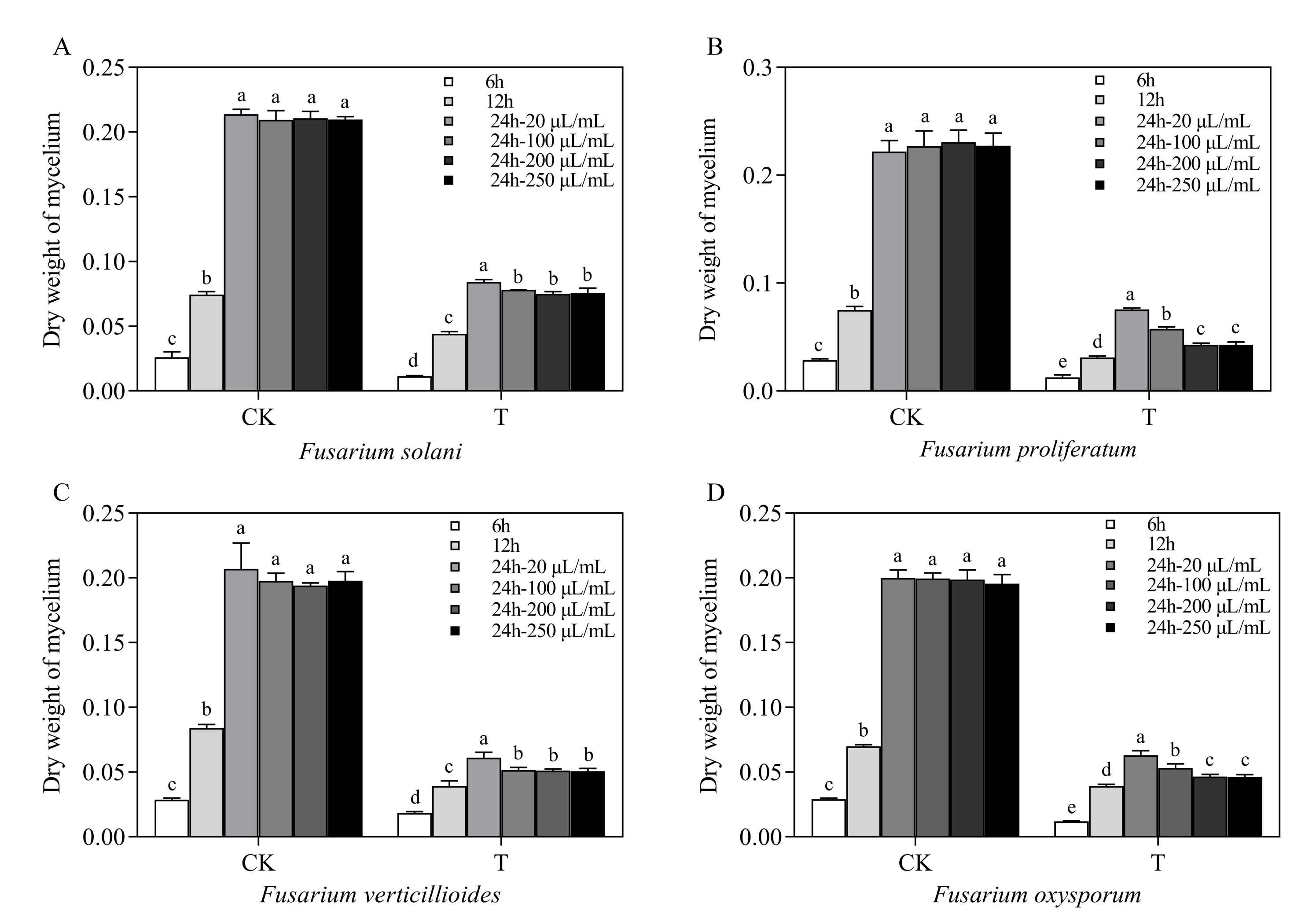
Figure S3:** Effect of fermentation broth on mycelial weight of pathogenic fungi. CK was mixed with sterile water and PDB liquid medium. T was mixed with fermentation broth and PDB liquid medium. Different letters indicate significantly different at 5 % level by Duncan’s new multiple range test. Values are mean ± SD.

**Figure S4:** The stability of cell-free culture filtrate. A Thermal stability, B UV sensitivity, C Acid-base stability, D Light stability. Different letters indicate significantly different at 5 % level by Duncan’s new multiple range test. Values are mean ± SD.


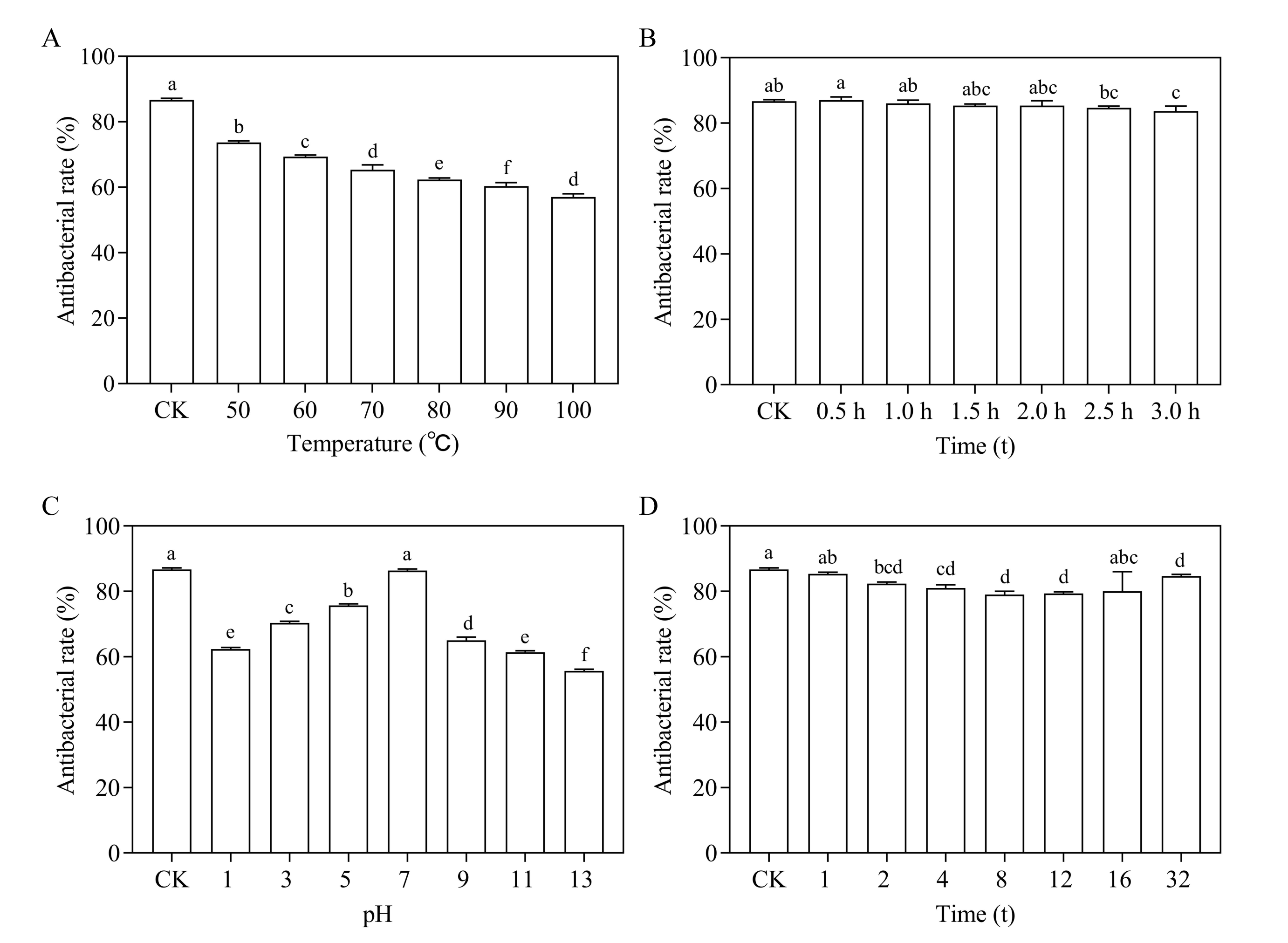


**Figure S5:** The result of vitro antibacterial test of each component. A *Aspergillus flavus*, B *Penicillium brasilianum*, C *Alternaria alternata*, D *Albifimbria verrucaria*, E *Fusarium solani*, F *Fusarium proliferatum*,G *Fusarium oxysporum*, H *Fusarium verticillioides*.


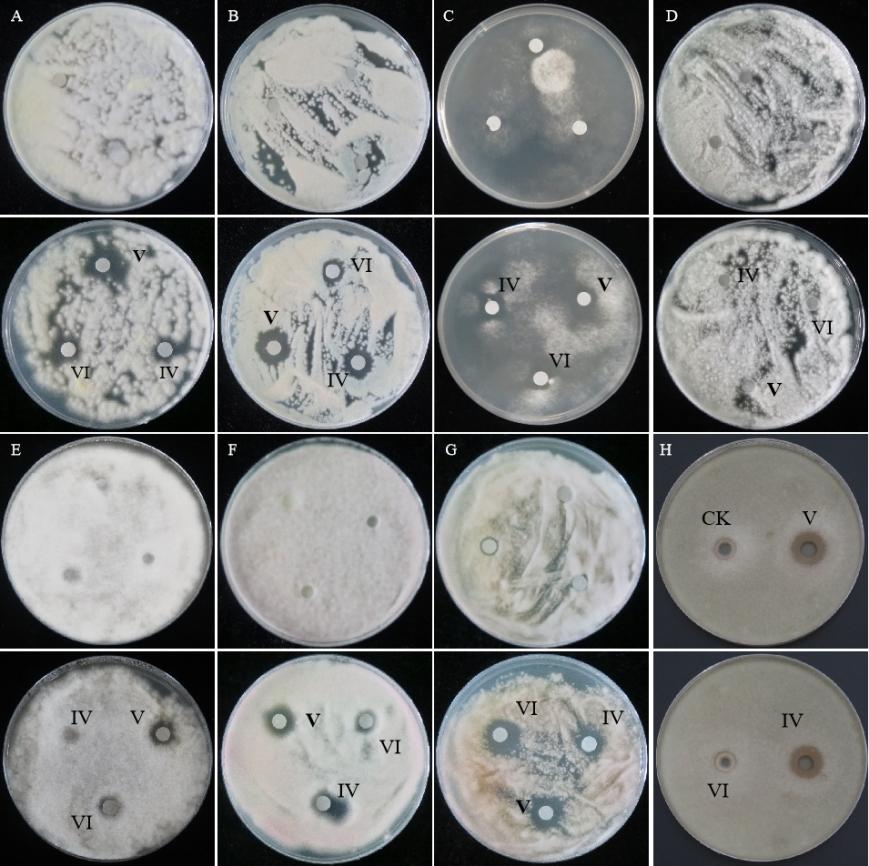


**Figure S6:** Mass spectrum of the representative compounds. Mass spectrum of the compound obtained by GC-MS analysis was compared with the NIST17 spectral database. If the ion fragment size and height similarity of the mass spectrum are above 95, the molecular formula, molecular weight and molecular structure of the substance can be determined. The abscissa represents the mass of the ion; the ordinate represents the intensity of the ion current. The left side was the mass spectrum of the compound, and the right side was the result after comparison with NIST17 spectral database. A Terephthalic acid, diisopropyl ester (GC RT 28.92, 30.022, 29.634 and 29.835 min); B 1,2-Benzenedicarboxylic acid (RT 22.28 and 23.058 min); C Isopropyl methyl phthalate (RT 26.132, 26.542 and 27.051 min); D Phthalic acid, 3,4-dimethylphenyl methyl ester (RT 27.647 min); E Benzeneacetic acid, 3-hydroxy-, methyl ester (RT 27.122, 26.117, 31.463 and 31.223 min); F Carbamic acid, (4-aminophenyl)-, methyl ester (RT 26.698 and 25.615 min); G Methyl 4-hydroxyphenylacetate, TMS derivative (RT 25.895 ); H sec-Butyl 3,5-dinitrobenzoate (RT 27.55 min).

G

F

E

D

C

B

A

H

**Figure S7:** The inhibitory effect of pure product on the growth of *Fusarium*. The left was the control (200 μL sterile distilled water), the right was the pure product (200 μL metabolite). A Terephthalic acid, diisopropyl ester, B 1,2-Benzenedicarboxylic acid, C Carbamic acid, (4-aminophenyl)-, methyl ester, D Benzeneacetic acid, 3-hydroxy-, methyl ester.

**
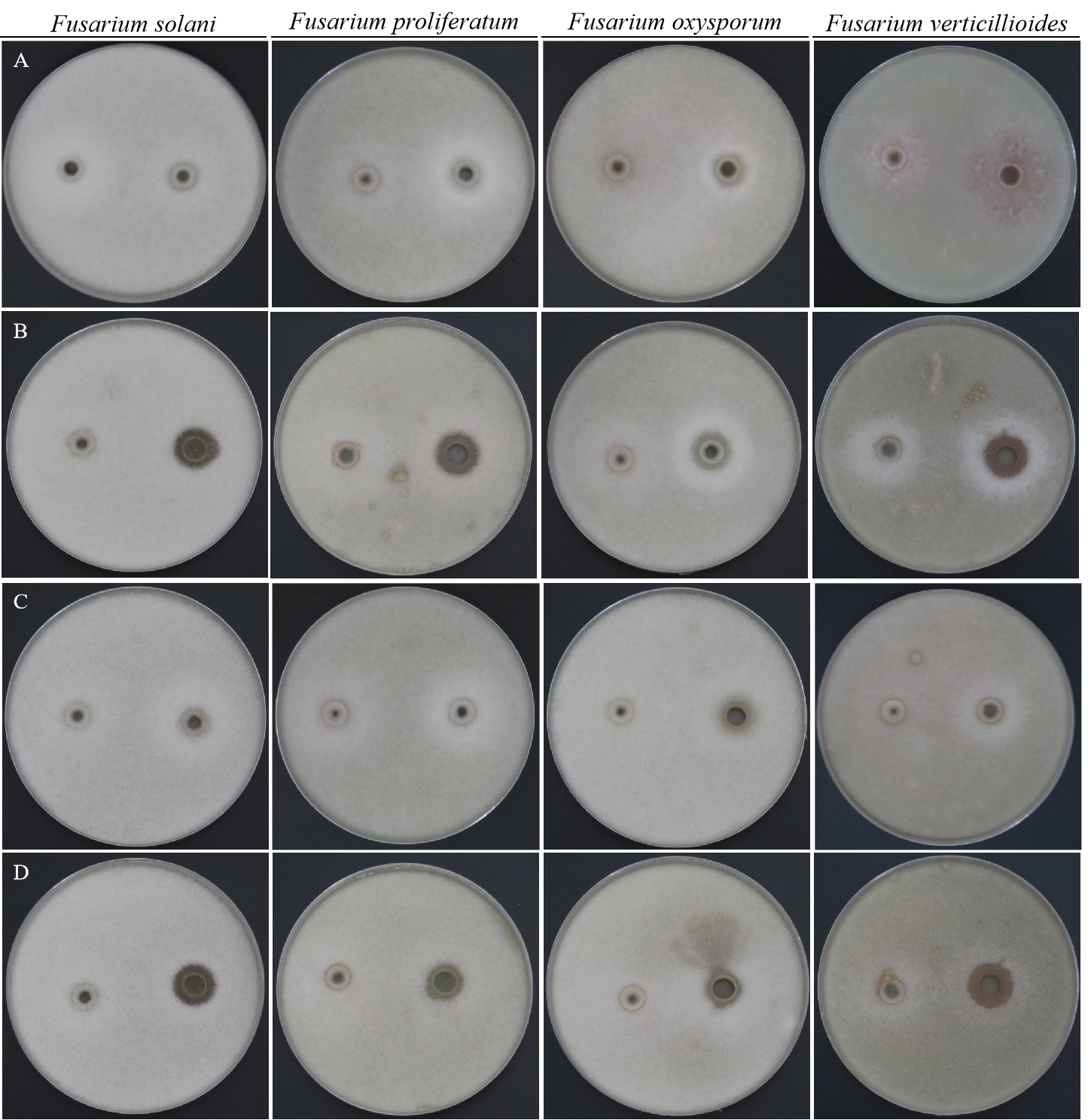
**

**Figure S8:** (A) Microscopic observation of the longitudinal section of the plant root. *Fusarium verticillioides* and *Fusarium oxysporum*: The roots of the plants were soaked with the conidia suspension for 12h. A-B, E-F Dense mycelium appeared in the epidermis of the root system, and the epidermal and cortical cells appeared ruptured, shedding and deformed (arrows), and the cells were arranged irregularly. D, G The conidia and hyphae of *Fusarium* appeared in the cortex and vascular column. C, H Viscous substances and starch granules appear in the cortex and vascular columns, which are densely accumulated on the cell wall. (B) Microscopic observation of the plant root stained by PAS. Mock Sterile distilled water treatment, FB fermentation broth treatment. A-L The roots of the plants were soaked with the conidia suspension of *Fusarium proliferatum* for 12h. Mock The root tissue was intact, the cell boundaries are clear and neatly arranged. FB The conidia and hyphae of *Fusarium proliferatum* were attached to the epidermis, the epidermis and cortex cells were slightly broken, deformed, and the internal tissue structure is intact. C, E-H Dense mycelium appeared in the epidermis of the root system, and the epidermal and cortical cells appeared ruptured, and deformed (arrows), and the cells were arranged irregularly. D Cauliflower-like structure appeared in the infected root areas (arrows). A-B Viscous substances and starch granules appear in the cortex and vascular columns, which are densely accumulated on the cell wall. I-L The conidia and hyphae of *Fusarium* appeared in the cortex and vascular column.


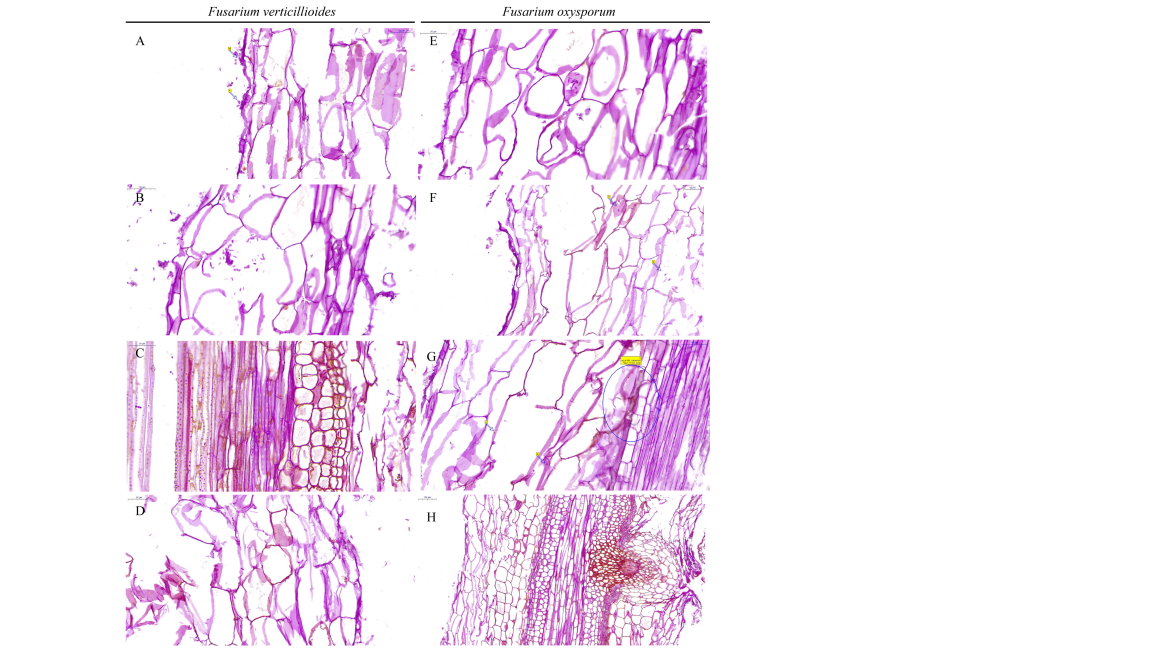


（A）

（B）


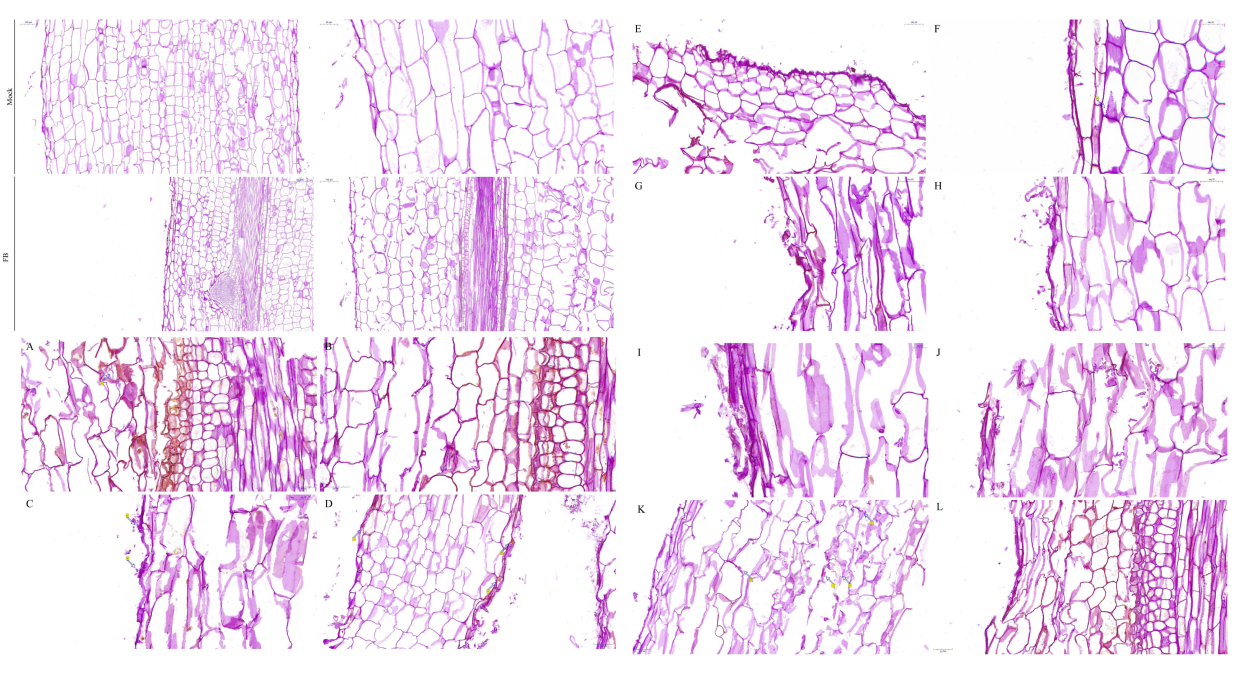


**Figure S9:** Growth promotion of Arabidopsis thaliana Col‐0 with exposure to pure product. (a) Effect of 1,2-Benzenedicarboxylic acid (B 100 μg) and Benzeneacetic acid, 3-hydroxy-, methyl ester (D 500 μg) on A. thaliana Col‐0 growth. Wa: Water, En: Ethanol. (b) Plant fresh weight, (c) Length of primary root, (d) Number of lateral roots (Primary lateral root and Secondary lateral root). Lowercase letters above the columns indicate a significant difference at p < 0.05. Values are mean ± SD.


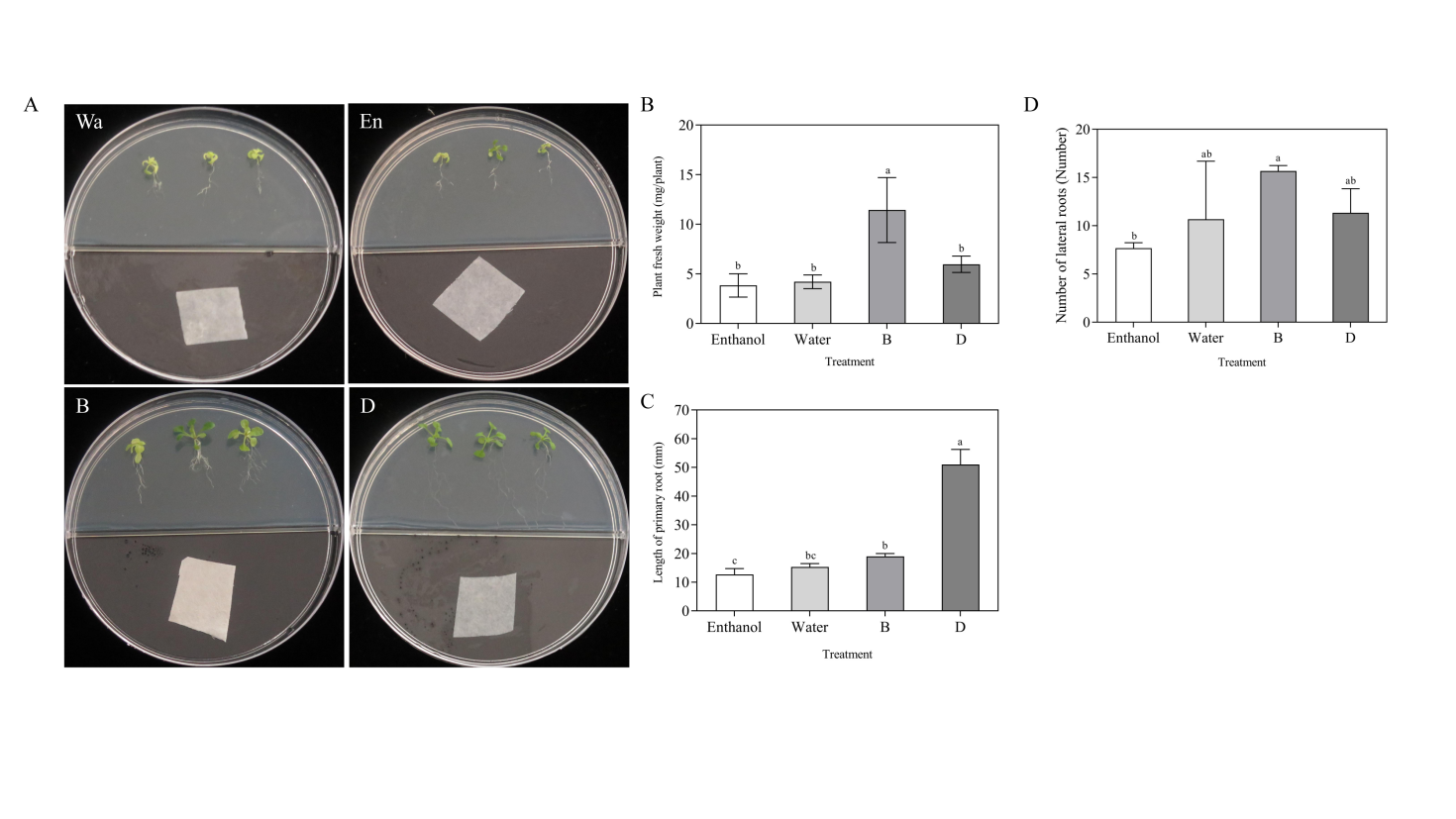


**Figure S10:** Total ion chromatogram of component Ⅳ and Ⅴ. The abscissa represents the peak time, and the ordinate represents the peak height. The figure is marked with retention time. Component Ⅳ only marked the components with Area% >1.0.


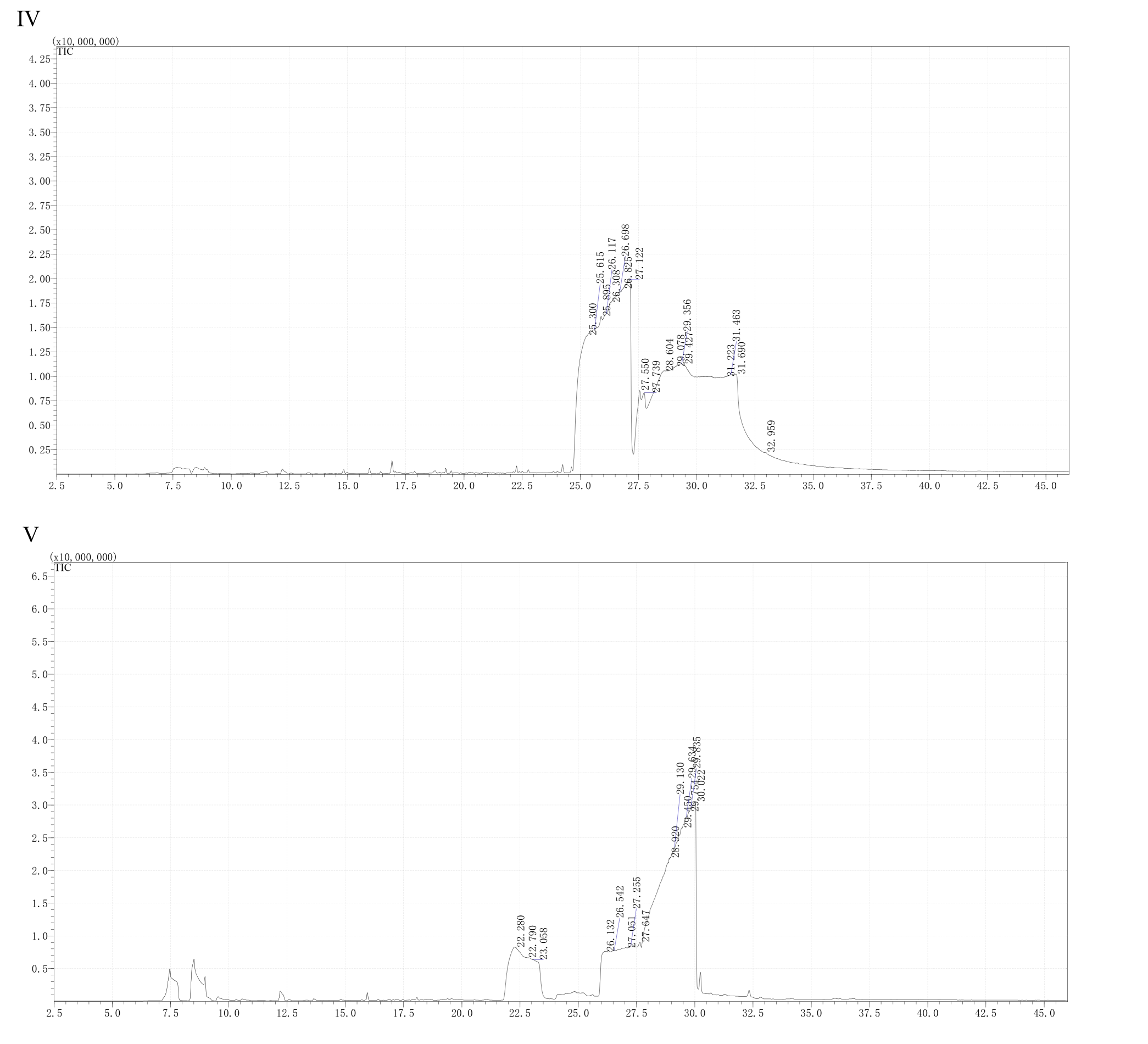

Supplement: Supplementary file 2 [file Data_Sheet_2.docx]
